# Physicochemical and Sensory Analysis of Sorghum, Rice, and Teff Flours Blending With Flaxseed Flour for Better Quality *Injera*

**DOI:** 10.1002/fsn3.4680

**Published:** 2025-01-10

**Authors:** Moges Amtataw, Estifanos Kassahun, Solomon Tibebu, Tadele Andargie, Takele Ayanaw, Agimassie Agazie, Mesfin Wogayehu, Abebaw Teshome, Sadik Jemal, Deginet Teferi

**Affiliations:** ^1^ Food, and Beverage Industry Research and Development Center Manufacturing Industry Development Institute Addis Ababa Ethiopia; ^2^ Innovation Incubation Center and Intellectual Property Right Coordination Office University‐Industry Linkage and Technology Transfer Directorate, Nanotechnology Center of Excellence; Addis Ababa Science and Technology University Addis Ababa Ethiopia; ^3^ Department of Environmental Engineering, College of Engineering, Sustainable Energy Center of Excellence, Bioprocess and Biotechnology Center of Excellence, Nanotechnology Center of Excellence Addis Ababa Science and Technology University Addis Ababa Ethiopia; ^4^ Faculty of Chemical and Food Engineering, Bahir Dar Institute of Technology Bahir Dar University Bahir Dar Ethiopia

**Keywords:** blending ratio, D‐optimal mixture design, gluten free, hedonic ratings

## Abstract

This study investigates the optimization of four gluten free flours namely sorghum, rice, teff flours, and 3% flaxseed flour blends to enhance the quality *injera*, which was traditionally baked with only pure teff. Utilizing a D‐optimal mixture design, ratios were varied (sorghum 43%–50%, rice 20%–27%, teff 23%–30%). Methods followed AOAC and AACC standards, analyzed using Minitab 19.2 software with one‐way and two‐way ANOVA. Results show flaxseed supplementation significantly improves sorghum‐based injera's texture and sensory attributes, approaching teff *injera* quality. Hedonic ratings (color, rollability, softness, taste, eye distribution, mouthfeel, and overall acceptability) were favorable. Physical texture remained stable during storage, with variable titratable acidity among blends. This research supports integrating flaxseed flour in grain blends to enhance *injera's* nutritional and sensory qualities, proposing applications in both household and industrial settings. The purpose of this study is to investigate the supplementation of flaxseed flour in a blend of sorghum, rice, and teff flours, for its performance to improve the overall acceptability, and sensory appeals of the produced *injera.*

## Graphical abstract

This study finds that adding 3% flaxseed flour to blends of sorghum, rice, and teff enhances the texture and sensory qualities of injera, making it comparable to traditional teff injera. The D‐optimal mixture design yielded favorable ratings in color, taste, and overall acceptability, while maintaining stable texture during storage. These results support the integration of flaxseed flour to improve injera's nutritional and sensory attributes for household and industrial applications.

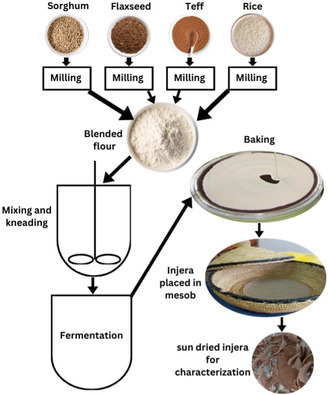

## 1 Introduction

*Injera*, the staple Ethiopian fermented flatbread, is made from Teff ( *Eragrostis Tef* ) flour. Teff is recognized for its superior nutritional value compared to common cereal grains like wheat, barley, sorghum, maize, and rice, primarily because it is used as a whole grain (Woldemariam et al. 2019a). Unlike animal‐based foods, cereal grains are deficient in proteins, fats, minerals, and vitamins, and they also lack some essential fatty acids found in oil seeds (Samtiya, Aluko, and Dhewa 2020; Kumar et al. 2022). There is a growing global interest in developing and marketing functional foods (Siró et al. 2008; Oniszczuk et al. 2020).

According to (Abewa and Abay 2020), excellent sensory outcomes and nutrient content were observed for the ratio of blending substitute to teff flour in both urban and rural communities. (Melaku 2022) also found that *injera* made from a blend of sorghum and teff improves the nutritional properties and sensory acceptability of *injera* while reducing the consumption of expensive teff. Though *injera* blended with other cereals like sorghum is often viewed as inferior, teff remains the most esteemed cereal in Ethiopia, both as a staple food and for its economic value (Wójcik et al. 2022). Sorghum, an energy‐rich cereal, contains complex carbohydrates like fibers and starches that digest slowly, providing satiety and delaying hunger (Mengistu et al. 2011). Achieving acceptable quality from the best varietal blends is essential for food and nutritional security among subsistence farmers (Moretti et al. 2014). Although *injera* making is a manual process, researchers have focused on improving composite flour for a better nutritional profile, the fermentation process can reduce anti‐nutritional factors like phytic acid, and increase mineral availability in *injera* other than sensory analysis (Samtiya, Aluko, and Dhewa 2020). According to Cherie et al. (2018), optimal formulation in terms of physical and compositional factors is important for selecting raw materials.

This research focuses on sorghum, rice, and flaxseed addition on teff for *injera* making. This food is a staple in Ethiopia because it can retain its palatability for more than 3 days. Nowadays, other cereals such as sorghum, maize, rice, wheat, and barley are also used to make *injera*, with sorghum being the second most preferred cereal for this purpose in Ethiopia (Melaku 2022). Sorghum (*Sorghum bicolor L. Moench*) is a crucial cereal in some semiarid regions of Africa, including Ethiopia and Niger. According to a 2019 CSA report of Ethiopia, sorghum production is estimated at 51.7 million quintals from 1.9 million hectares, with an average yield of 27.26 quintals per hectare. Sorghum accounts for 14.96% of the total grain‐producing area, cultivated by over 5 million farmers, (Umwungerimwiza 2014; Ghebrehiwot et al. 2016) study notes limited research on improving sorghum *injera*‐making technology. Rice (*Oryza sativa L*.) is a staple for over 50% of the global population, particularly in Asia. Rice consumption in Ethiopia has increased significantly, reflecting a broader trend across Africa (Balasubramanian et al. 2007; Tadele 2017). Some Ethiopians blend rice flour with teff flour to make *injera*, favoring its lighter color and excellent expansion properties due to its high starch content. Rice flour also enhances *injera*'s soft texture, taste, elasticity, and whiteness, and is more cost‐effective than teff flour is valued for its health benefits and nutritional content. It improves dough uniformity and elasticity, reduces staling, and enhances the nutritional value of baked goods (Yoseph et al. 2018; Attuquayefio and Assefa 2019). Flaxseed‐enriched cereal products also show better processing conditions without significant lipid oxidation (Oniszczuk et al. 2019; Mitrus et al. 2020). Substituting up to 9% teff flour with flaxseed flour in *injera* increases its functional properties, offering enhanced omega‐3 fatty acids, dietary fiber, and antioxidants. Flaxseed incorporation could thus improve the nutritional profile of *injera*, a staple in Ethiopia (Mercier et al. 2014; Saha et al. 2024).

People frequently choose to include cereals, such as those previously mentioned, which are easily accessible, budget‐friendly, and inexpensive for both meal preparation and generating income. However, using sorghum flour for *injera* presents challenges, particularly with textural hardness. Research on improving the quality of sorghum *injera* is limited (Abewa and Abay 2020; Ghebrehiwot et al. 2020). Sorghum *injera* tends to lose its freshness, softness, and rollability and becomes brittle and dry during storage, which are significant issues. Staling, which includes various chemical and physical texture changes but not microbial spoilage, reduces consumer acceptance of sorghum *injera* (Melaku 2022). This process results in sorghum *injera* becoming harder, drier, and more friable. The staling is primarily due to moisture transfer from the crumb to the crust and the firming of the cell wall material caused by starch re‐crystallization during storage (Joseph et al. 2012). Due to these factors, the preparation of *injera* requires mixture optimization to enhance its physicochemical properties and sensory acceptance. This study was conducted to explore the supplementation of flaxseed flour in a blend of sorghum, rice, and teff flours, for its ability to enhance the overall acceptability, color, softness, Instrumental texture, Titratable acidity, roll ability, taste, mouthfeel, and eye distribution of *injera*.

## 2 Materials and Methods

### 2.1 Sample Collection

The following experimental materials were used. White sorghum (*Melkam* variety) were collected from *Gonder* Agricultural Research Center, brown rice (*X‐Jigena* variety) from *Fogera* National Rice Research and Training Center, white *Teff Qoncho Teff* variety (DZ‐Cr‐387), and flaxseed (*Geregera* Variety) from *Adet* Agricultural Research Center. Varieties were selected based on their popularity, intensive production, and common usage by Ethiopian farmers.

### 2.2 Sample Preparation

**FIGURE 1:**
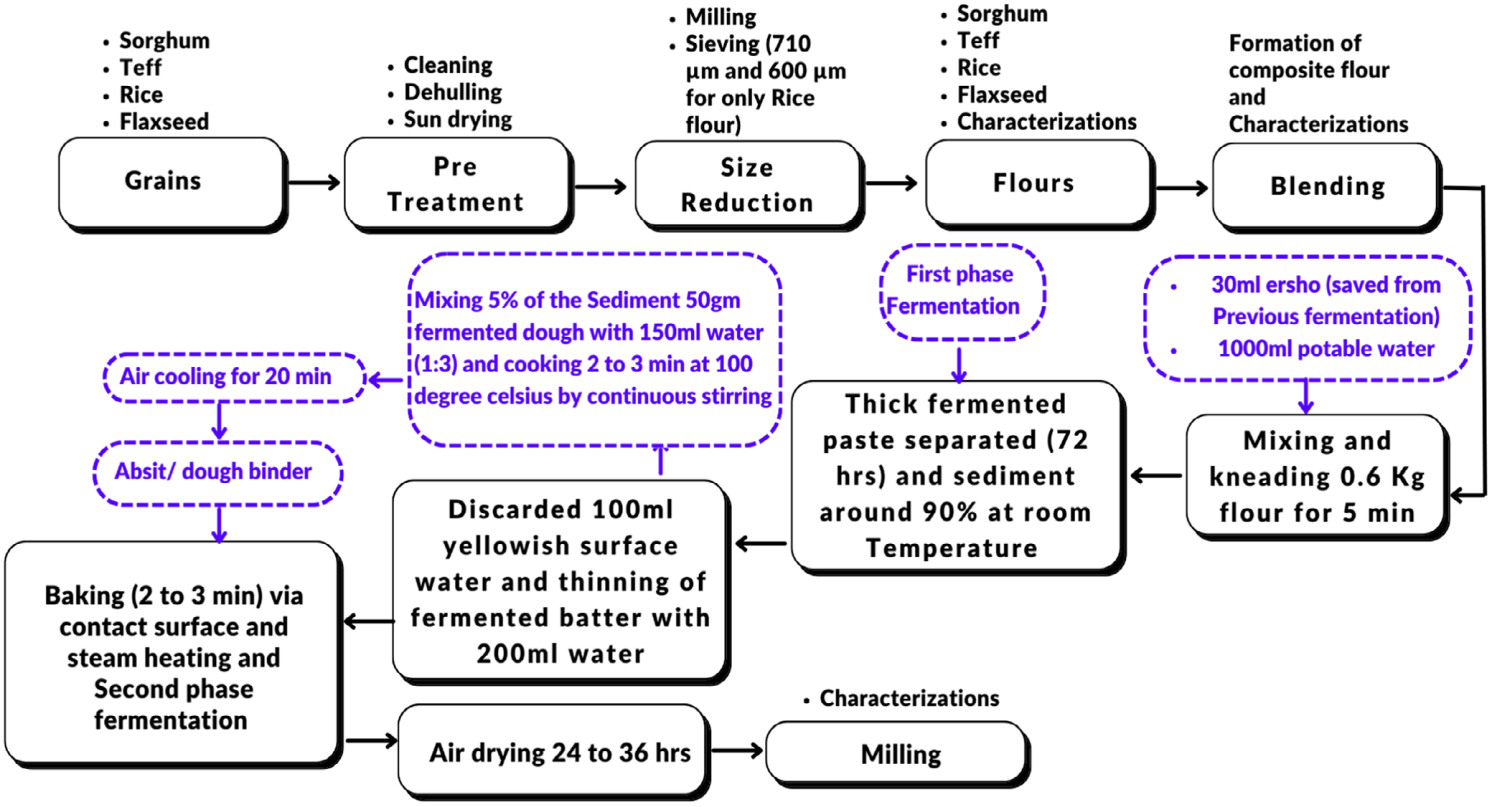
The overall experimental framework of the study design.

The collected samples were prepared according to reported studies (Girma, Bultosa, and Bussa 2013; Yoseph et al. 2018). Each ingredient was prepared according to the procedure shown in Figure 1. The Sorghum, Rice, Teff, and Flaxseed Grains were manually cleaned by winnowing and handpicking. The Sorghum, rice, and teff grains were milled by a community cottage disk miller to whole flour and sieved by 710 μm and 600 μm sieve sizes based on their effectiveness (Girma, Bultosa, and Bussa 2013). Flaxseed is also milled or ground using a high‐speed multi‐function comminutor oilseed and coffee mill machine. The sample was packed in a polyethylene plastic bag at refrigeration temperature (4°C) until further experimental analysis.

### 2.3 Treatment Combinations and Experimental Design

The experiment was conducted by the D‐optimal mixture experimental design with three independent variable ingredients. The D‐optimal mixture design is utilized in experiments involving the combination of one or more substances, creating a new experimental area within a subregion of the mathematically feasible proportion space.

The blending ratios of sorghum, rice, and teff flours were set based on preliminary trial work and previous studies from the literature (Girma, Bultosa, and Bussa 2013; Cherie et al. 2018) and generated by using Minitab software version 19.2. The 3% flaxseed flour was constantly added for each experimental treatment except for 100% sorghum and teff flour treatments. Lower and upper ranges of treatment combinations were Sorghum flour (43%–50%), Rice flour (20%–27%), and Teff flour (23%–30%) respectively for the lower and upper limit of ingredients (Woldemariam et al. 2019b; Melaku 2022).

### 2.4 Composite Flour Preparation

The composite flour was prepared according to the generated experimental design for *injera* preparation. The *injera* was prepared with 100% sorghum flour and 100% teff flour considered as the control (Yetneberk 2004; Yetneberk et al. 2004; Yetneberk, Rooney, and Taylor 2005). The composition is shown in Table 1.

**TABLE 1:** Formulations of blending ratios with constant supplemented flaxseed flour.

Sample Code	Blocks	Sorghum flour (%)	Rice flour (%)	Teff flour (%)	Supplement flaxseed flour (%)
Con1	1	100	0	0	0
Con2	1	0	0	100	0
BR1	1	50	27	23	3
BR2	1	50	20	30	3
BR3	1	46.5	23.5	30	3
BR4	1	46.5	27	26.5	3
BR5	1	43	27	30	3
BR6	1	47.67	24.67	27.66	3
BR7	1	45.34	25.83	28.83	3
BR8	1	48.83	22.34	28.83	3
BR9	1	48.83	25.83	25.34	3
BR10	1	50	23.5	26.5	3

Abbreviations: BR = blending ratio, con1 = 100% sorghum flour, con2 = 100% teff flour.

### 2.5 Batter Preparation, Fermentation, and *Injera* Preparation

The *injera* was made by specified composite flour 0.6 kg (600 g) with 1 litter water (1000 mL) to the thick dough, and the fermentation was initiated by adding 30 mL of *Ersho*, a starter culture which was left and saved from the preliminary test batter (Yetneberk 2004; Yetneberk et al. 2004; Abewa and Abay 2020). Then the thick dough was kneaded by hand for 5 min to maintain dough kneading uniformity. After finished kneading, the kneading vessel was closed and allowed 72 h for first‐stage fermentation at room temperature. Subsequently, before thinning the dough 100 mL liquid layer that typically forms over the dough is gently poured off and thinned dough with 200 mL potable water. After that, *Absit* was prepared by taking the fermented dough batter from the first stage of fermentation. To prepare absit, 5% of fermented dough was mixed with potable water ratio (1:2) and cooked at 100°C for 3 min to gelatinize the starch. Then, the gelatinized batter (*Absit*) was cooled to room temperature and added back to the fermenting dough.

The second fermentation phase was carried out for 16 h, the bubble formation indicates the endpoint of the fermentation reach and ready to bake. Finally, 100 mL of potable water was added to the fermented dough to bring correct batter consistency (Bultosa 2007; Yoseph et al. 2018; Abewa and Abay 2020). About 500 g of fermented batter was poured in a circular manner motion from the outer perimeter towards the center, onto a hot‐round smooth baking griddle called *Metad* (onto a 45–50 cm diameter hot clay griddle). The *Metad* is covered with a *Metad* lid called *Akambalo* (made from bamboo, grass, and mud) to prevent steam from escaping. Before pouring the batter, the *Metad* surface was rubbed with the rapeseed flour using a piece of cloth. Finally, the *Injera* was baked at a temperature of 190°C–210°C for 2–3 min. The baked *Injera* was removed from the *Metad* and kept in an airtight container *Mesob* (a traditional storage facility made of woven grass straw).

### 2.6 Determination of Functional Properties of Flour

#### 2.6.1 Bulk Densities of Raw Materials Flour

The bulk density of sorghum, rice, teff, and flaxseed flours was determined by the method of (Awol, Kuyu, and Bereka 2023). Around 100 g flour samples were taken into a 100 mL measuring cylinder (tube) and tapped several times on a laboratory bench to a content volume. The volume of the sample was recorded and the bulk density of the sample from the values obtained using Equation (1):

(1) $$\text{Bulk density}\left(\frac{g}{\mathrm{ml}}\right)=\frac{\text{sample of weight}}{\text{volume of the sample after tapping}}$$

#### 2.6.2 Water Absorption Capacity of Raw Material Flour

Water absorption capacity (WAC), which indicates the amount of water available for gelatinization, was determined according to the method used by (Emmanuel, Osuchukwu, and Oshiele 2010). Exactly 2.5 g of each sample was added to 25 mL distilled water in a weighed 50 mL centrifuge tube. The tube was agitated for about 5 min in before being centrifuged at 3000 rpm for 30 min. The mixture was decanted and the clear supernatant was discarded. Then the non‐bounded drops of water were carefully drained off as much as quantitatively possible and the tube was reweighed. The following Equation (2) was used for the determination of WAC.

(2) $$\mathrm{WAC}\;\left(\%\right)=\frac{\text{weight of water}-\text{bound}}{\text{weight of the sample}\;\left(\mathrm{dry}\;\text{basis}\right)}\times 100$$

#### 2.6.3 Water Absorption Index and Water Solubility Index

The water absorption index (WAI) and water solubility index (WSI) of flour were determined as described by (Chauhan and Singh 2013). The 2.5 g flour sample was dispersed into 30 mL of distilled water using a glass rod and heated at 90°C for 15 min in a water bath. The cooked paste was cooled to room temperature and transferred to a tare centrifuge tube and centrifuged at 3000 rpm for 10 min. The supernatant was transferred into a tare evaporating dish for determination of dry solid content by evaporating the supernatant overnight at 105°C then the sediment was weighed. WAI and WSI were calculated by Equations (3 and 4).

(3) $$\mathrm{WAI}\;\left(\frac{g}{g}\right)=\frac{\text{weight of sediment}}{\text{weight of flour}}$$

(4) $$\mathrm{WSI}\;\left(\%\right)=\frac{\text{weight of dissolved solids in the supernatant}}{\text{weight of flour sample}}\;x\;100$$

#### 2.6.4 Determination of Composite Flour Pasting Properties

The composite flour sample was analyzed by a rapid Visco analyzer (RVA). Around 3.5 g of mixed flour was weighed (weight‐adjusted to 14% moisture basis) as determined by the American Association of Cereal Chemists (AACC) International method 76–21.01, ICC Standard No. 162, and 25 distilled water was blended. After blending, the flour‐water slurry was transferred to the RVA. The RVA was set at 50°C as the starting temperature and held at the same temperature for 1.5 min. Later, the slurry was heated to 95°C at the rate of 10°C per minute while being maintained for 2 min at peak temperature. The paste viscosity properties of the composite flour were examined peak viscosity (PV), final viscosity (FV), breakdown viscosity (BDV), setback viscosity (SBV), peak temperature (PT), and peak time (Pt). All viscosity values were recorded in centipoise (cP). The peak temperature (PT) in degrees centigrade (°C) and peak time (Pt) minute (min) were also recorded (Kuo, Hong, and Thseng 2001; Raina et al. 2007). The measurements were done in triplicate.

### 2.7 Determination Proximate Composition of *Injera* Products

#### 2.7.1 Moisture Content

The moisture content of raw material sorghum, rice, and teff flour sample in dry base (DB) and formulated composite flour freshly‐baked *injera* product in wet base (WB) was determined by oven drying method according to Association of Official Agricultural Chemists AOAC (2000) method number 930.15. The moisture dish was cleaned and dried in an oven at 105°C for 1 h and placed in desiccators to cool. The weight of the blank moisture petri dish (W_1_) was determined initially. Then 5 g samples (in triplicate) were taken in the dried moisture petri dish (W_2_) at 105°C for 6 h until constant weight and after being cooled in desiccators to room temperature, it was again weighed (W_3_). Then, the moisture content was estimated by Equation (5).

(5) $$\text{Moisture content}\;\left(\mathrm{MC}\%\right)=\frac{W2-W3}{W2-W1}\times 100$$

#### 2.7.2 Total Ash

The official method 942.05 of AOAC (2000) was applied to calculate ash content. The mass of the crucible was measured by analytical balance (M_1_). About 5 g of the sample was weighed into crucibles (M_2_). The sample was then placed in a furnace at about 550°C until free from carbon and the residues appeared grayish white (about 8 h). The sample was removed from the furnace placed into the desiccators and weighed (M_3_), the ash content was analyzed by following Equation (6).

(6) $$\text{Total}\;\mathrm{ash}\;\left(\%\right)=\frac{M3-M1}{M2-M1}\times 100$$

#### 2.7.3 Crude Protein

Protein content was determined according to the Kjeldahl method of crude protein analysis 920.87 methods for flour (AOAC 2000). About 0.5 g of food sample was weighed on an analytical balance into the digestion flask or larger test tube. Then the sample was digested between 50°C and 415°C temperature for 190 min with the addition of a small volume (5 mL) of concentrated H_2_SO_4_ (an oxidizing agent that digested the food), anhydrous Na_2_SO_4_ that sped up the reaction by raising the boiling points of H_2_SO_4_ and a catalyst CuSO_4_ to speed the reaction. About 1 g of catalyst mixture was made of Na_2_SO_4_ with anhydrous CuSO_4_ in the ratio of 10:1. Digestion has converted any nitrogen in the food (other than that which is in the form of nitrates or nitrites) into ammonia and other organic matter to CO_2_ and H_2_O. In an acidic solution, ammonia was not liberated as gas because rather it exists as the ammonium sulfate salt. After digestion was completed, the content in the flask was diluted with 50 mL distilled water and 40 mL NaOH (40%) solution was added to the Sample to liberate ammonia gas and immediately attached to the distiller. The ammonia was then distilled into a receiving flask that consisted of 25 mL of boric acid solution (4%) for reaction with ammonia until 150 mL after that the solution was from the distiller. Boric acid was used, and the borate ion was titrated with standard acid (0.1 N HCL).

(7) $$\text{Total nitrogen}=\frac{\left(T-B\right)*N*14.007*100}{W}$$

Where: T‐Volume in mL of the standard acid solution used in the titration for the test material, *B*—Volume in mL of the standard acid solution used in the titration for the blank determination, *N*—Normality of standard sulfuric acid, and *W*—Weight in grams of the test material

(8) Crude protein=conversion factor*total nitrogen

Conversion factor (5.7) for sorghum, rice, and teff cereals and (5.3) for flaxseed (WHO and FAO 2007).

#### 2.7.4 Total Carbohydrate

The total carbohydrate content of the samples was determined by subtraction of the parameters of the above test from 100% (AOAC 2000).

(9) $$\mathrm{CHO}\;\left(\%\right)=100\%-\left(\%M+\%\mathrm{CP}+\%A+\%\mathrm{CF}+\%\mathrm{CF}\right)$$

Where; % M (DB)—percent of moisture content in dry base, %CP—percent of crude protein, %A—percent of ash, %CF‐ percent of crude fiber, and %CF—percent of crude fat.

### 2.8 Sensory Evaluation

**FIGURE 2:**
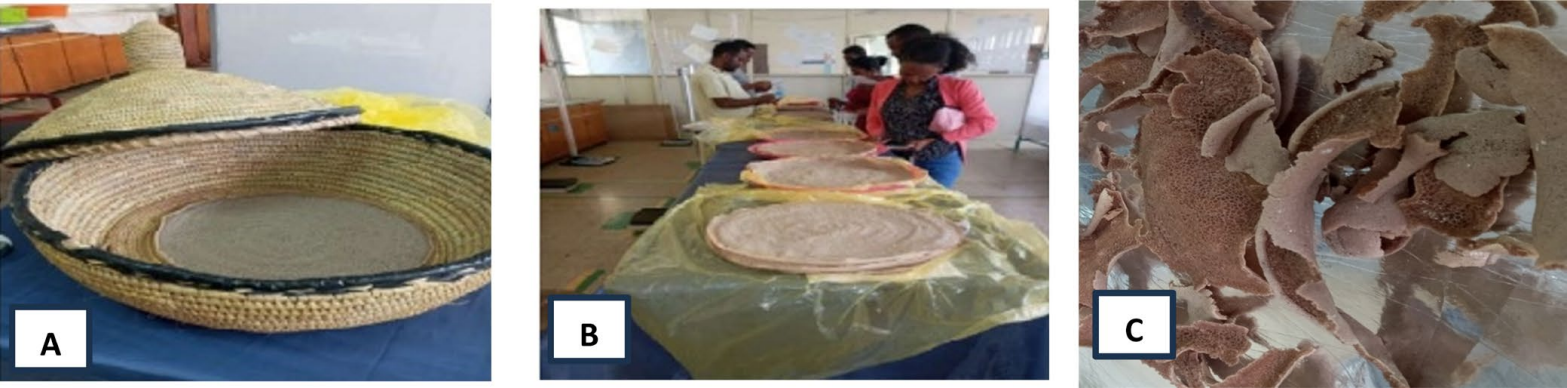
The baked *injera* shown on (A), Sensory evaluation formulated injera using a five‐point hedonic scale (B) and sun *injera* (C).

The 10 formulated *injera* samples and two controls (100% sorghum and 100% teff) were prepared and subjected to sensory evaluation. The sensory evaluation was carried out based on color, softness, rollability, taste, eye distribution, mouthfeel, and overall acceptability using a five‐point hedonic scale, where 1—dislike extremely, 2—dislike moderately, 3—neither like nor dislike, 4—like moderately and 5—like extremely (Pimentel, Gomes da Cruz, and Deliza 2015). A total of 47 untrained consumer panelists were randomly selected from *Bahir Dar* town's residents, *Bahir Dar* Institute of Technology staff members, and students. Panelists were almost equally distributed in gender and age ranging from 20 to 50. Freshly prepared sorghum‐based *injera* was served on a randomly coded plate and was evaluated within 2 h after baking. Individual evaluator panelists have assessed the *injera* for taste, mouthfeel, color and texture, size, evenness, and distribution of gas holes (honey crumb eyes) on the upper surface and smoothness of the bottom surface of the *injera*. During the evaluation, panelists used water for palate cleaners in between each sample's sensory analysis. Figure 2 depicts the sensory assessment of formulated injera utilizing a five‐point hedonic scale.

### 2.9 Physiochemical Properties of *Injera*

#### 2.9.1 pH Of Freshly‐Baked *Injera*

About 10 g of *injera* sample was mixed with 100 mL potable boiled distilled water, and the supernatant was then decanted into a 250 mL beaker, and immediately pH of each homogenate was determined using a glass electrode attached to a pH meter (AOAC 1995; Nielsen 1998).

#### 2.9.2 Titratable Acidity

Titratable acidity (TA) was measured by titration of a 5 g sample in 45 mL of 0.1 N NaOH solution using 0.1 mL 0.5% phenolphthalein as an indicator and then the percent of lactic acid present in the sample was calculated using a formula (Obi, Wang, and Brown 2018);

(10) $$\text{Lactic acid}\;\left(\%\right)=\frac{\text{amount of NAOH}\;X\;\text{normality of NAOH}\times 9}{\text{the volume of the sample in}\;\mathrm{ml}}$$

#### 2.9.3 Instrumental Color (L* Value) Characteristics Formulated *Injera*

A Hunter Lab color spectrophotometer colorimetry SP‐CM 600D (Konica Minolta Inc., made in Japan) was employed to measure the color of the food flour samples. A White and black standard tile was used for calibration as standard reference reading (L, a, b). The color values are expressed as L* (Whiteness or Darkness), a* (Redness or Greenness), and b* (Yellowness or Blueness). For the color analysis, the flour samples are packed in a transparent zip bag and placed against the light source to measure the color. Three measurements are analyzed for each substitution level and are analyzed at three different locations (Basics and Color 2001). The color difference (∆E) is calculated by applying Equation (11).

(11) $$\mathrm{\Delta E}={\left[{\left(L*-L\;\right)}^{2}+{\left(a*-a\;\right)}^{2}+{\left(b*-b\;\right)}^{2}\;\right]}^{1/2}$$

The whiteness index (WI) values that combine lightness and yellow‐blue into a single term is calculated as follows:

(12) $$\mathrm{WI}=100-{\left[{\left(100-L\right)}^{2}+a^{2}+b^{2}\;\right]}^{1/2}$$

#### 2.9.4 Instrumental Texture Analysis

Textural storage properties of *injera* (day‐1, day‐2, and day‐4 storage) hardness were determined using a TA‐XT^2^ texture analyzer (Stable Micro Systems, Godalming, UK), with a flat‐ended cylindrical probe by the method (Abang Zaidel et al. 2008). After baking, *injera* samples were allowed to cool and stored for about 0, 2, and 4 days at room temperature (about 25°C). The thickness of the fresh and stored *injera* strips was measured using calipers. The testing profile was as follows: pre‐test speed (1.0 mm/s), test speed (3.0 mm/s), post‐test speed (10.0 mm/s), distance (15 mm), and trigger type (0.049 N, Auto). The sample strips were placed over the vertical struts (30 mm apart) of the bending rig and clamped in place at both ends. The strips were compressed at a constant rate of 10 mm/s over a distance of 15 mm. The peak force (*N*) of each composite flour *injera* was measured by the hardness cutting of bending force.

### 2.10 Statistical Data Analysis and Optimal Validation

All the experimental data measurements were made in triplicated except microbial load which was duplicated, and the data were analyzed using Minitab software version 19.2 statistical method analysis (linear and quadratic model regression for mixtures, one‐way ANOVA for raw material and one‐factor analysis, two‐way ANOVA for two‐factor analysis or repeated measures of general linear model). The statistical mean values and significance differences of the parameters examined were done using Tukey's pairwise comparison with a significant level at 5% (*p* < 0.05).

## 3 Result and Discussion

### 3.1 Proximate Composition of Raw Material Flours

The percentage of proximate composition of the ingredients (sorghum, rice, and teff flours), along with the supplement flaxseed flour used for preparing *injera*, is presented in Table 2. The moisture content of teff flour (8.20%) and sorghum flour (8.27%) showed no significant difference. However, these values significantly differed (*p* < 0.05) from rice flour (9.73%) and flaxseed flour (5.53%). The moisture content of sorghum flour was consistent with (Woldemariam et al. 2019b), who reported 7.1%. Similarly, the moisture contents for teff flour (8.20%), flaxseed flour (5.53%), and rice flour (9.73%) were consistent with the findings of (Bultosa et al. 2019) (Bultosa and Taylor 2004).

**TABLE 2:** Proximate composition of raw ingredients analysis.

Component Variable	Moisture Content (%)	Total Ash (%)	Crude Fat (%)	Crude Fiber (%)	Crude Protein (%)	Total Carbohydrate (%)	Energy /Calorific Value (Kcal/100 g)
Sorghum flour	8.27 ± 0.25^b^	1.57 ± 0.26^b^	3.81 ± 0.10^b^	4.63 ± 0.03^c^	9.00 ± 0.10^b^	72.73 ± 0.28^b^	361.20 ± 0.92^b^
Rice flour	9.73 ± 0.25^a^	1.44 ± 0.11^b^	1.62 ± 0.07^d^	5.07 ± 0.18^b^	7.18 ± 0.30^d^	74.96 ± 0.31^a^	343.08 ± 0.68^d^
Teff flour	8.20 ± 0.27^b^	2.24 ± 0.11^a^	2.05 ± 0.06^c^	4.35 ± 0.03^d^	8.01 ± 0.09^c^	75.12 ± 0.42^a^	350.97 ± 1.92^c^
Flaxseed flour	5.53 ± 0.06^c^	1.06 ± 0.11^c^	36.54 ± 0.77^a^	21.73 ± 1.12^a^	18.25 ± 0.12^a^	16.89 ± 0.21^c^	469.39 ± 0.57^a^

*Note:* The values are presented in mean ± standard deviation of triplicates, and all parameters were measured on a dry basis (% db.) content. The alphabetic symbols 2 represent statistical groupings, specifically used to indicate significant differences in the proximate composition of the different raw ingredients. These letters correspond to the results of statistical tests (such as ANOVA), which compare the means of the parameters (Moisture Content, Total Ash, Crude Fat, etc.) for each ingredient. (a), (b), (c), and (d) indicate which groups of ingredients are statistically similar or different. For example: b (e.g., for Sorghum flour Moisture Content: 8.27 ± 0.25) means that Sorghum flour is statistically different from the ingredient(s) with symbol a, but it is not significantly different from the ingredient(s) with symbol b. a (e.g., for Rice flour Moisture Content: 9.73 ± 0.25) means Rice flour is statistically different from the ingredient(s) marked with b, c, or d for that specific component. Similarly, c and d represent different statistical groupings, showing which ingredients have similar or significantly different values.

The total ash content, which reflects the mineral composition of the food materials, ranged from 1.44% to 2.24%. Rice had the lowest total ash content (1.44%), which was not significantly different (*p* < 0.05) from sorghum flour (1.57%) but significantly different (*p* < 0.05) from teff flour (2.24%) and flaxseed flour (1.06%). Teff flour is known for its mineral richness, containing up to 3.14% total ash, as reported by (Bultosa 2007) and (Baye 2014), which aligns with the current findings. Rice flour's ash content (1.7%) also matches the report by (Islam et al. 2012). Sorghum flour's total ash content was reported to range from 1.3% to 3.30% which is in agreement with the results of (Makokha et al. 2002). Crude fat is a crucial energy source, enhancing the palatability of food by absorbing and retaining aromas and flavors. The crude fat contents of sorghum, rice, teff, and flaxseed flours were 3.81%, 1.62%, 2.05%, and 36.54%, respectively, with significant differences (*p* < 0.05) observed among the flours.

The crude fiber content of the flours ranged from 4.35% to 21.73%. Specifically, the crude fiber content was 4.63% for sorghum, 4.35% for teff, 21.73% for flaxseed, and 5.07% for rice flour. These values align with findings from (Makokha et al. 2002; Bultosa 2007; Devi et al. 2013), respectively. The mean grouping of crude fiber content showed significant differences (*p* < 0.05). The protein content for sorghum, rice, teff, and flaxseed flours was 9.00%, 7.18%, 8.01%, and 18.25%, respectively, consistent with reports from (Makokha et al. 2002; Meherunnahar et al. 2018). There were highly significant differences (*p* < 0.05) in protein content among the flours.

The carbohydrate content in sorghum, rice, teff, and flaxseed flours was 72.73%, 74.96%, 75.12%, and 16.89%, respectively. According to (Baye 2014), teff contains 80% complex carbohydrates and about 73% starch, classifying it as a starchy cereal. The carbohydrate content of teff and rice did not significantly differ, but both were significantly higher than that of sorghum and flaxseed flour. The gross energy content of the samples varied significantly, with rice at 343.08 kcal/100 g, teff at 350.97 kcal/100 g, sorghum at 361.20 kcal/100 g, and flaxseed at 469.39 kcal/100 g, showing significant differences (*p* < 0.05). The high‐fat content in flaxseed significantly influenced the overall energy values in the blended product.

### 3.2 Functional Properties of Raw Material Flour

#### 3.2.1 Bulk Density

The Bulk Density (BD) of teff, sorghum, rice, and flaxseed flours were 0.78, 0.82, 0.86, and 0.67 g/ml^3^, respectively, and presented in Table 3. According to (Guo et al. 2013), bulk density decreases linearly when moisture content decreases in the dry base (DB). The moisture content of teff, sorghum, rice, and flaxseed flour are 8.20, 8.27, 9.73%, and 5.53%. The result shows that the moisture content of rice was comparatively high as that of sorghum and teff the bulk density also linearly increased. The result has been agreed with (Aregbesola, Adedeji, and Ajibola 2015; Olagunju et al. 2018; Melaku 2022) report. The variation in bulk density of flour could be due to the variation in starch content. The higher the flour starch contents the more likely the increase in bulk density. The increased bulk density of flours suggests their suitability for application in food preparations. In contrast, low bulk density would be useful in the formulation of complementary foods [56].

**TABLE 3:** Functional properties of raw material flour.

Components	Bulk Density (g/ml^3^)	Water Absorption Capacity (%)	Water Solubility Index (%)	Water Absorption Index (g/g)
Sorghum flour	0.82 ± 0.01^b^	173.87 ± 5.74^b^	4.25 ± 0.25^b^	2.74 ± 0.06^b^
Rice flour	0.86 ± 0.01^a^	163.24 ± 2.71^b^	2.59 ± 0.23^c^	2.63 ± 0.06^b^
Teff flour	0.78 ± 0.01^c^	139.83 ± 6.33^c^	4.70 ± 0.18^b^	2.40 ± 0.03^c^
Flaxseed flour	0.67 ± 0.01^d^	216.22 ± 5.86^a^	10.42 ± 0.93^a^	3.16 ± 0.06^a^

*Note:* Each mean value ± standard deviation of triplicates. The alphabetic symbols indicate statistical groupings for each functional property (Bulk Density, Water Absorption Capacity, Water Solubility Index, Water Absorption Index) across the different flour types (Sorghum, Rice, Teff, and Flaxseed). (a) represents the highest or distinct group for that functional property. (b)–(d) represent subsequent groups where values are significantly similar within the same group but different from other groups.

#### 3.2.2 Water Absorption Capacity

The water absorption capacity (WAC) of sorghum, teff, rice, and flaxseed flours, observed through gelatinization, was recorded as 173.8%, 139.83%, 163.24%, and 216.22%, respectively, as shown in Table 3. Teff flour had the lowest WAC at 139.83 g/100 g, while flaxseed flour had the highest at 216.22 g/100 g. The WAC of sorghum and rice flours did not significantly differ (*p* < 0.05). Flaxseed flour's WAC was notably higher than the other three constituents of *injera* composites. The higher WAC in components may be attributed to their higher protein content and soluble fiber (Morris 2007), as well as the loose structure of starch polymers. Conversely, a low WAC value indicates a more compact molecular structure (Muthia, Nurul, and Noryati 2010), which can affect the dryness, final attributes, and shelf life of the baked *injera* (Zghal, Scanlon, and Sapirstein 2001; Iwe, Onyeukwu, and Agiriga 2016). Variations in WAC results might be due to differences in protein concentration, water interaction, and conformational characteristics (Tadesse, Bultosa, and Abera 2019).

#### 3.2.3 Water Solubility Index and Water Absorption Index

The water solubility index (WSI) for sorghum, rice, teff, and flaxseed starch was 4.25%, 2.59%, 4.70%, and 10.42%, respectively. Flaxseed's higher solubility can be attributed to its high amylose content, which dissolves in hot water (*p* < 0.05). Significant differences (*p* < 0.05) in WSI were observed between flaxseed, sorghum, teff, and rice flours, though teff and sorghum did not significantly differ. The WSI of rice flour (2.59%) aligns with the findings of (Cornejo and Rosell 2015).

The water absorption index (WAI) for sorghum, rice, teff, and flaxseed starch was 2.74 g/g, 2.4 g/g, 2.63 g/g, and 3.16 g/g, respectively. The WAI result for teff flour is consistent with the (Attuquayefio and Assefa 2019) report, which found an average WAI of 2.65 g/g for teff flour. Variations in WAI indicate the extent to which the internal starch structure in the flour is exposed to water (Collar 2003). Flaxseed flour exhibited the highest WAI (3.16 g/g) among the flours, likely due to its high protein content, which contains subunits that dissociate upon heating, providing more water‐binding sites, as observed by (Tadesse, Bultosa, and Abera 2019). Protein subunits increase the number of hydrophilic groups, the primary sites for water binding. (Inglett, Chen, and Lee 2013) noted that fiber content, particle size, and mineral and protein content can enhance the water‐binding capacity of flours, affecting hydration properties through interaction and coagulation. Flours with lower hydration capacity require more flour to make dough compared to those with higher hydration capacity. The swelling and absorption index of flours is influenced by temperature and the types of composite flour starches.

### 3.3 Pasting Properties of Composite Flour

#### 3.3.1 Peak Viscosity

As shown in Table 4, the peak viscosity (PV) of composite flours ranged from 1366.0 to 2187.5 cp. The peak viscosity of individual sorghum, teff, and rice flours was 1449 cp, 2070 cp, and 2164.5 cp, respectively. Significant differences (*p* < 0.05) in peak viscosity were observed between the control flours (100% sorghum, rice, and teff) and most composite flours, except for BR4 and BR5 among samples with different blending ratios. The highest peak viscosity (2187.5 cp) was found in BR8 (48.83% sorghum, 22.34% rice, 28.83% teff), while the lowest (1366.0 cp) was in BR1 (50% sorghum, 27% rice, 23% teff). This indicates that higher PV corresponds to greater swelling power, whereas lower PV suggests higher solubility due to starch degradation or dextrinization (Mohammed, Mustafa, and Osman 2009). Additionally, the thickening ability and water‐holding capacity of the pasted composite flours contribute to the softness and rollability of *injera*, compared to 100% sorghum flour. The increased composition of rice and teff flours affects the PV of composite flours for *injera* quality, aligning with reports by (Ndie, Nnamani, and Oselebe 2010; Tadesse, Bultosa, and Abera 2019; Abebaw Tsegaye and Abera 2020). Despite the constant flaxseed flour supplementation in each composite flour, it enhances starch granule hydration by bonding with available water, thereby reducing the staling factor of *injera*.

**TABLE 4:** Pasting properties of composite flour measurement with rapid Visco analyzer (RVA).

Composition	Peak viscosity (cP)	Breakdown viscosity (cP)	Final viscosity (cP)	Setback viscosity (cP)	Peak temperature (°C)	Peak time (min)
CON1	1449.0 ± 15.0^f^	478.0 ± 9.0^g^	2149.5 ± 125.5^c^	1287.0 ± 128.0^b^	73.83 ± 0.4^a^	5.24 ± 0.0^d^
CON2	2070.0 ± 14.0^de^	1207.5 ± 16.5^a^	2903.5 ± 33.5^ab^	1932.5 ± 39.5^a^	64.38 ± 0.0^cde^	5.37 ± 0.0^bcd^
CON3	2164.5 ± 30.5^ab^	1151.0 ± 29.0^bc^	3192.0 ± 22.0^a^	2178.5 ± 20.5^a^	63.98 ± 0.5^de^	5.54 ± 0.1^a^
BR1	1366.0 ± 8.0^g^	566.5 ± 3.5^f^	2242.5 ± 10.5^c^	1443.0 ± 6.0^b^	68.10 ± 0.4^b^	5.34 ± 0.1^cd^
BR2	2139.0 ± 18.0^abc^	1126.0 ± 4.0^bcd^	3105.0 ± 9.0^a^	2092.0 ± 13.0^a^	67.25 ± 0.5^bc^	5.44 ± 0.0^abc^
BR3	2074.0 ± 19.0^cde^	1097.0 ± 12.0^cde^	2954.5 ± 111.5^a^	1978.0 ± 104.5^a^	66.85 ± 0.1^bcd^	5.37 ± 0.0^bcd^
BR4	2116.5 ± 25.5^bcd^	1059.0 ± 37.0^e^	3138.0 ± 14.0^a^	2080.5 ± 25.5^a^	67.35 ± 0.4^b^	5.50 ± 0.1^ab^
BR5	2113.5 ± 3.50^bcd^	1160.0 ± 19.0^bcde^	3113.0 ± 10.0^a^	2107.5 ± 4.0^a^	62.63 ± 3.4^e^	5.47 ± 0.0^abc^
BR6	2044.0 ± 44.0^e^	1053.5 ± 2.5^e^	2514.0 ± 459.0^bc^	1523.0 ± 417.0^b^	66.95 ± 0.0^bc^	5.44 ± 0.0^abc^
BR7	2134.0 ± 11.0^abcd^	1121.0 ± 6.0^bcd^	3038.0 ± 78.0^a^	2025.0 ± 73.0^a^	66.90 ± 0.0^bc^	5.50 ± 0.0^ab^
BR8	2187.5 ± 18.5^a^	1106.0 ± 3.0^ab^	3122.5 ± 14.50^a^	2095.0 ± 15.0^a^	66.90 ± 0.0^bc^	5.44 ± 0.0^abc^
BR9	2086.5 ± 38.5^cde^	1073.0 ± 33.0^de^	3109.0 ± 19.0^a^	2095.5 ± 13.5^a^	66.85 ± 0.1^bcd^	5.44 ± 0.0^abc^
BR10	2100.0 ± 5.0^bcde^	1086.5 ± 13.5^de^	3120.5 ± 14.5^a^	2105.0 ± 16.5^a^	67.03 ± 0.1^bc^	5.44 ± 0.0^abc^

*Note:* Each value means ± standard deviation of triplicates. The alphabetic symbols are used to indicate statistical groupings for the pasting properties (Peak Viscosity, Breakdown Viscosity, Final Viscosity, Setback Viscosity, Peak Temperature, and Peak Time) of the different flour compositions. (a) represents the group with the highest values for a given property. (b)–(g) show progressively lower or similar values for the measured pasting properties.

Abbreviations: BR, blending ratio (1 up to 10); CON_1_, 100% sorghum flour; CON_2_, 100% teff flour; CON_3_, 100% rice flour, cp, centipoise.

#### 3.3.2 Final Viscosity

As shown in Table 4, the final viscosity ranged from 2242.5 to 3138.0 cp, with a significant difference (*p* < 0.05) observed between control flours and composite flours. The highest final viscosity (3138.0 cp) was found in BR4 (46.5% sorghum, 27% rice, 26.5% teff), while the lowest (2242.5 cp) was in BR1 (50% sorghum, 27% rice, 23% teff). The results indicated that increasing the proportions of teff and rice flour reduced the rate of starch retrogradation, thereby improving the quality of the composite flour *injera*. Although there was no comparison involving a 3% flaxseed flour supplement for each 100% of sorghum, rice, and teff flours in the blending ratios, the constant 3% flaxseed flour supplement did not significantly increase viscosity during heating compared to the control and composite flours. This lack of significant impact could be due to the small substitution amount. These viscosities are crucial for assessing the quality and ability of *injera* or food samples, particularly regarding retrogradation or re‐crystallization of soluble starch granule amylose during cooling and storage (Chinma, Abu, and Ojo 2010).

#### 3.3.3 Breakdown Viscosity

The measured breakdown viscosity (BDV) of the composite flours is presented in Table 4. The range of BDV results of composite flour values ranged from 566.5 to 1160.0 cp. The highest (1160 cP) and the lowest (566.5 cP) BDV values have been observed in BR5 (43% sorghum, 27% rice, 30% teff) and BR1 (50% sorghum, 27% rice, 23% teff) respectively. The breakdown viscosity of 100% sorghum flour (478 cp) < 100% rice flour (1151 cp) < 100% teff flour (1207.5 cp). This implies that teff flour, followed by rice flour, influences the breakdown viscosity of composite flour, impacting product quality. As a result, BR5 exhibits the highest disintegration (indicating low retrogradation) of the swollen systems and aligns the amylase components to enhance injera texture. In the case of composite flour (BR1), the breakdown viscosity reflects the stability of starch paste reassociation, leading to staling during processing and storage, beginning at the cooling stage.

The higher the viscosity breakdown, the lower the ability of the starch in the flour and composite flour samples to withstand heating and shear stress (Adebowale et al. 2011). It was also reported that a high breakdown value indicates relative weakness of the swollen starch granules against hot shearing while a low breakdown value indicates that the starch in question possesses cross‐linking properties (Anberbir et al. n.d.). The teff flour starch pasting properties are shear tolerant and thus had the potential for use in *injera* processed under high shear conditions. The breakdown is caused by the disintegration of the gelatinized starch granule structure during continued stirring and heating.

#### 3.3.4 Setback Viscosity

The setback viscosity (SBV) of composite flour ranged from 1443 to 2107 cP. The highest SBV (2107 cP) was observed in BR5 (43% sorghum, 27% rice, 30% teff), while the lowest (1443 cP) was in BR1 (50% sorghum, 27% rice, 23% teff). For 100% single flours, the SBV values were 1287 cP for sorghum, 1932.5 cP for teff, and 2178.5 cP for rice. Rice and teff flours significantly impacted the setback viscosity of composite flour *injera* during storage. The results indicated that BR5 had lower retrogradation during cooling and a slower staling rate in the formulated *injera*. Teff starch, known for its lower thickening ability, shear tolerance, and slow setback compared to sorghum starch (Yetneberk 2004), showed similar results in the RVA test. The significantly higher SBV of rice (2178.5 cP) and teff (1932.5 cP) compared to sorghum (1287 cP) is related to amylose retrogradation, suggesting that teff flour has a lower extent of retrogradation than sorghum. This lower retrogradation in teff flours could be beneficial for *injera* and other food products (Tadesse, Bultosa, and Abera 2019; Abewa and Abay 2020). High amylose content is believed to contribute to the absence of a peak, high stability during heating, and high setback during cooling. Significant differences (*p* < 0.05) were noted among the blending ratios, with synergistic effects observed. According to (Lipilina and Ganji 2009), flaxseed flour reduced the staling rate of sorghum‐based *injera*, even though a constant supplementation was added to each composite flour blend, increasing its setback viscosity during storage.

#### 3.3.5 Peak Temperature

The result of the peak temperatures (PT) of the composite formulated flour doughs ranged from 62.63‐to 68.10°C. Initially, components sorghum, teff, and rice flours as control gelation PT 73.83°C, 64.38°C, and 63.98°C, respectively with significant (*p* < 0.05) differences among them. The highest peak temperature (68.10°C) was observed in BR1(50% sorghum, 27% rice, 23% teff) and the lowest PT (62.63°C) was also observed in BR5(43% sorghum, 27% rice, 30% teff). The peak temperature of the composite flour is more affected by sorghum flour (73.83^0^ C) > teff flour (64.38^0^ C) > rice flour (63.98^0^ C). Higher peak temperature also indicates a greater structural rigidity of the flour and friable during storage leading to staling of sorghum *injera* limitation. The Peak temperature found in this study was similar to the report of (Tadesse, Bultosa, and Abera 2019) (64.1°C and 90.8°C) for flour starches. Supplement flaxseed flour affects each blended ratio pasting properties of peak temperature constantly, due to its constant incorporation.

#### 3.3.6 Peak Time

The peak time value of composite flour ranged from 5.34–5.50 min and for 100% sorghum, teff, and rice flour values were 5.24,5.37, and 5.54 min, respectively. There were significant (*p* < 0.05) differences between each flour. The highest Peak time (5.5 min) was observed in BR4 (46.5% sorghum, 27% rice, 26.5% teff) and BR7 (45.34% sorghum, 25.83% rice, 28.83% teff) and the lowest Peak time (5.34 min) also observed in BR1 (50% sorghum, 27% rice, 23% teff). Results show that a slightly short peak time observed in sorghum flour<teff flour<rice flour, might be due to reduced starch content and indicative of its ability to cook fast.

### 3.4 Mineral Content of Raw Material Flour

The iron, zinc, calcium, magnesium, manganese, and potassium mineral content of composition flours were presented in Table 5. The iron compositions of sorghum, teff, rice, and flaxseed flour were found to be 5.3, 15.04, 3.6, and 7.50 mg/100 g, respectively. Among compositions, teff flour produced the highest increase in iron content followed by flaxseed, sorghum, and rice, respectively, and showed a high significant difference (*p* < 0.05). The result of an iron content agreement with the previous researchers for sorghum (0.9–20 mg/100 g) (Makokha et al. 2002). The calcium content of sorghum, teff, rice, and flaxseed flour is 1.43,1.79, 2.34, and 9.04 mg/100 g, respectively. There was a non‐significant (*p* < 0.05) difference between sorghum, rice, and teff flour but a high significance (*p* < 0.05) difference in flaxseed flour.

**TABLE 5:** The mineral content of raw material flour (Each means value ± standard deviation of triplicates).

Components	Fe content (mg/100 g)	Zn content (mg/100 g)	Ca content (mg/100 g)	Mg content (mg/100 g)	Mn content (mg/100 g)	K content (mg/100 g)
Sorghum flour	5.37 ± 1.15^b^	1.99 ± 0.77^a^	1.43 ± 0.31^b^	4.74 ± 0.11^b^	1.63 ± 0.14^d^	37.32 ± 0.14^b^
Teff flour	15.04 ± 2.50^a^	2.58 ± 0.02^a^	1.79 ± 0.00^b^	4.88 ± 0.05^b^	5.80 ± 0.29^a^	40.85 ± 0.09^a^
Rice flour	3.60 ± 0.25^b^	2.69 ± 0.02^a^	2.34 ± 0.00^b^	4.81 ± 0.03^b^	3.45 ± 0.01^b^	34.91 ± 0.13^c^
Flaxseed flour	7.50 ± 4.24^b^	2.57 ± 0.01^a^	9.04 ± 0.62^a^	6.86 ± 0.06^a^	2.60 ± 0.08^c^	41.28 ± 0.30^a^

*Note:* The alphabetic symbols indicate statistical groupings for the mineral content of the raw material flours (Sorghum flour, Teff flour, Rice flour, and Flaxseed flour). These symbols were derived from the results of statistical analyses (such as ANOVA), which compare the mineral content across the different flour types. (a) typically indicates the group with the highest values for a particular mineral. (b)–(d) represent other groups, where values are significantly different or similar based on the statistical analysis.

The Zinc mineral content in sorghum flour (1.99 mg/100 g), rice flour (2.69 mg/100 g), teff flour (2.58 mg/100 g), and flaxseed flour (2.57 mg/100 g), respectively. The magnesium content of sorghum, rice, teff, and flaxseed flour was individually found that 4.74, 4.81, 4.88, and 6.86 mg/100 g, respectively. The mean value of each component of flour has a non‐significant difference (*p* < 0.05) except flaxseed flour. The manganese content of sorghum, rice, teff, and flaxseed flour constituted were 1.63, 5.80, 3.45, and 2.60 mg/100 g, respectively. There was a significant difference (*p* < 0.05) between each flour composition. Teff flour is a relatively high manganese content source for the product. The potassium content of sorghum, rice, teff, and flaxseed flour constituted were 37.32, 34.91, 40.85, and 41.28 mg/100 g, respectively. Teff and flaxseed flour do not have a significant (*p* < 0.05) difference in potassium content.

### 3.5 Physiochemical Properties of Baked *Injera*

#### 3.5.1 pH Value of Fresh *Injera*

**FIGURE 3:**
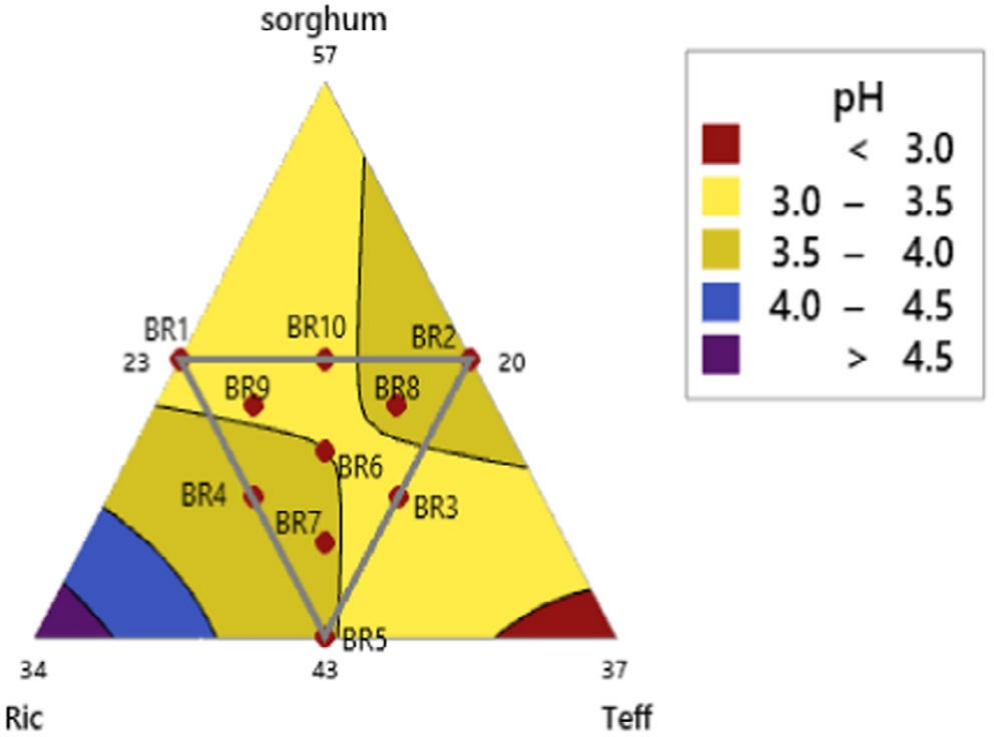
Mixture Contour Plot of pH.

The pH and titratable acidity are key physicochemical properties that indicate the sourness of *injera* is presented in Table 6. In this study, the pH and titratable acidity of *injera* made from different composite flour compositions were significantly different (*p* < 0.05). The pH values of sorghum composite flour fermented dough *injera* ranged from 3.34 to 3.76. The highest pH value (3.76) was observed in BR1 (50% sorghum, 27% rice, 23% teff), and the lowest (3.34) in BR2 (50% sorghum, 20% rice, 30% teff). The mixture contour plot (Figure 3) showed that increasing the sorghum composition decreased the pH value of *injera*. The pH readings taken immediately after baking varied slightly among the blending ratios. Another study indicated that the pH value depended on the lactic acid content in the fermented batter on the day of baking and decreased as fermentation time increased (Wendy 2014). The moisture and starch content of the starting materials significantly affect the pH value during fermentation due to the amount of fermentable sugars present. (Wendy 2014) reported an inverse relationship between pH and moisture content in commercially available *injera*, with pH values ranging from 3.65 (60.40% moisture) to 4.02 (44.46% moisture). Various literature sources report different pH readings for *injera* due to factors such as fermentation time, removal of supernatant liquids, and the amount of back‐slope starter culture (*Ersho*) used. The pH is influenced by the type of flour used, with rice flour having the most significant impact, followed by sorghum flour, as shown in the mixture contour plot (Figure 3) and model Equation (12). Cereal flour with a pH of 5.0 to 6.2, rich in fermentable carbohydrates, is preferred for fermentation by lactic acid bacteria, typically reducing the pH to around 3.4. The Ethiopian Standard Agency specifies the pH of teff *injera* to be between 3.45 and 4.0 (ES 3788:2018).

**TABLE 6:** Physicochemical properties of freshly baked injera, and Instrumental texture (hardness cutting force) analysis result.

Component variable	pH	Titratable acidity	Texture analysis (hardness, cutting force) (N)		
			Day‐1 storage	Day‐2 storage	Day‐4 storage
CON1	3.33 ± 0.07^e^	0.11 ± 0.01^d^	12.74 ± 0.84^a^	15.60 ± 0.79^a^	15.78 ± 1.08^a^
CON2	3.76 ± 0.03^a^	0.17 ± 0.00^a^	2.96 ± 1.63^c^	5.60 ± 0.41^b^	5.96 ± 0.47^b^
BR1	3.72 ± 0.03^ab^	0.11 ± 0.01^cd^	7.60 ± 0.39^b^	7.65 ± 0.54^b^	7.67 ± 0.46^b^
BR2	3.34 ± 0.01^e^	0.12 ± 0.01^cd^	7.10 ± 0.37^b^	7.19 ± 0.26^b^	7.31 ± 0.89^b^
BR3	3.43 ± 0.04^de^	0.14 ± 0.02^bc^	6.40 ± 0.56^b^	6.43 ± 0.81^b^	6.53 ± 0.71^b^
BR4	3.60 ± 0.03^bc^	0.12 ± 0.02^bcd^	6.40 ± 0.42^b^	6.44 ± 0.96^b^	6.74 ± 0.53^b^
BR5	3.55 ± 0.06^cd^	0.15 ± 0.02^ab^	6.20 ± 0.23^b^	6.25 ± 0.72^b^	6.46 ± 0.39^b^
BR6	3.54 ± 0.04^cd^	0.12 ± 0.01^bcd^	6.90 ± 0.38^b^	6.92 ± 1.04^b^	6.97 ± 0.62^b^
BR7	3.54 ± 0.05^cd^	0.14 ± 0.01^bc^	6.30 ± 0.64^b^	6.31 ± 0.84^b^	6.48 ± 0.64^b^
BR8	3.51 ± 0.05^cd^	0.14 ± 0.01^bc^	7.10 ± 0.68^b^	7.18 ± 1.14^b^	7.26 ± 0.59^b^
BR9	3.48 ± 0.04^d^	0.12 ± 0.01^cd^	7.01 ± 0.65^b^	7.11 ± 0.33^b^	7.22 ± 0.56^b^
BR10	3.44 ± 0.04^de^	0.12 ± 0.01^bcd^	7.56 ± 0.55^b^	7.59 ± 0.82^b^	7.67 ± 0.61^b^

*Note:* The alphabetic symbols are used to represent statistical groupings for the physicochemical properties (pH, Titratable Acidity) and the instrumental texture analysis (hardness, cutting force) of freshly baked injera during storage. These groupings were derived from the results of statistical analyses (such as ANOVA), which compare the values for each parameter across the different formulations and storage days. (a) represents the group with the highest or lowest value for a specific parameter. (b)–(e) represent other groups, where values are statistically similar within the same group but significantly different from other groups with different letters.

The predicted pH value is estimated accordingly by Equation (12) and its coefficient of determination (*R* ^2^ = 96.72%).

(12) $$\begin{array}{c} \mathrm{pH}\;\text{value}=-0.035202*\text{sorghum}+0.907935*\text{rice}–0.558932\\ {}*\text{teff}–0.015045*\text{sorghum}*\text{rice}+0.015011*\text{sorghum}\\ {}*\text{teff}–0.005656*\text{rice}*\text{teff} \end{array}$$

#### 3.5.2 Titratable Acidity Value of Fresh *Injera*

**FIGURE 4:**
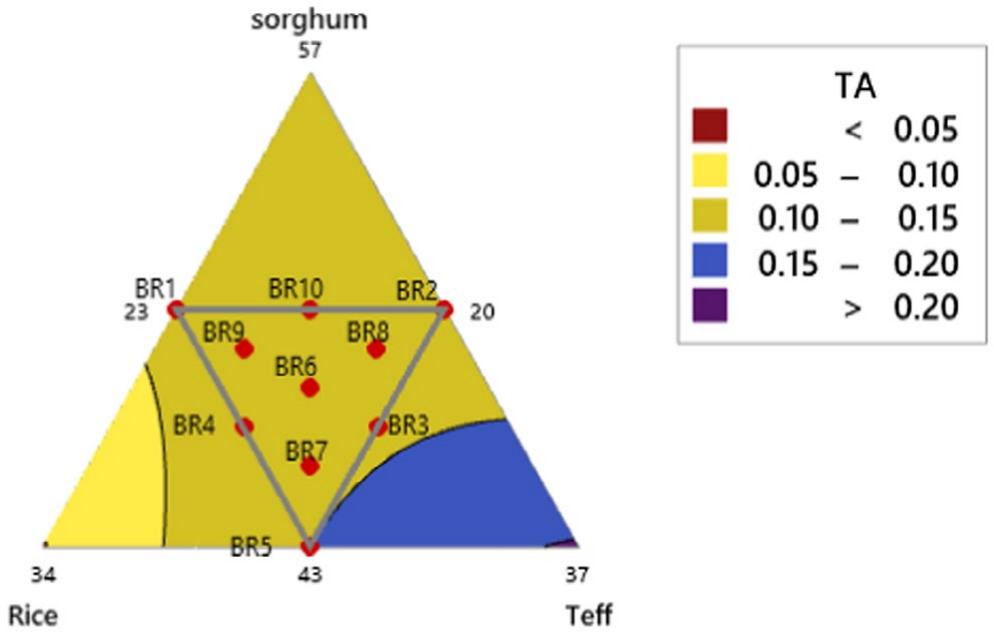
Titratable Acidity (TA) interaction of baked injera.

The titratable acidity (TA) of the composite formulated *injera* and the two control *injeras* made from 100% sorghum and 100% teff ranged from 0.11 to 0.15 and 0.11 to 0.17, respectively. The highest TA was observed in BR5 (43% sorghum, 27% rice, 30% teff), while the lowest was in BR1 (50% sorghum, 27% rice, 23% teff). The results indicate that increasing the proportion of teff flour leads to a linear increase in TA values (*R* ^2^ = 86.34%), followed by sorghum flour, as shown in Equation (13). The constant addition of flaxseed flour equally affected the TA of each blended composite flour *injera*. The sourness of traditionally fermented Ethiopian *injera*, influenced by pH changes due to lactic acid bacteria during fermentation, was also noted by, those who reported a pH value of 3.83 with increased fermentation time (Yigzaw et al. 2004). Figure 4 demonstrates the interaction of titratable acidity (TA) in baked injera.

In other cases, rice and teff composition directly increases the pH value of composite flour fresh‐baked *injera*.

(13) $$\begin{array}{cc} \mathrm{TA}= & 0.0087155*\text{sorghum}–0.0388121*\text{rice}+\\ & 0.0313601*\text{teff}+0.0004935*\text{sorghum}*\text{rice}–\\ & 0.0008441*\text{sorghum}*\text{teff}+0.0004918*\text{rice}*\text{teff} \end{array}$$

The result of sorghum flour control *injera* pH value (3.33) is less than from other composite flour *injera* products and teff control *injera* pH value (3.76). In the following mixture contour plot, as teff composition increases pH value directly decreases and TA increases. Whereas, as rice composition increases the result shows vice versa of composite flour.

#### 3.5.3 Instrumental Texture Properties of Baked *Injera* at Different Storage Periods

The textural properties of sorghum‐based *injera* are critical for its physical acceptability, particularly concerning hardness or compressed cutting force. The physical texture values for composite flour *injera*, measured with a texture analyzer, ranged from 6.20 to 7.60 N, 6.25 to 7.65 N, and 6.46 to 7.67 N on days 1, 2, and 4 of storage, respectively. For sorghum and teff flour *injera* used as controls, the texture values on days 1, 2, and 4 of storage were 12.74 N, 15.60 N, and 15.78 N for sorghum, and 2.96 N, 5.60 N, and 5.96 N for teff. The highest texture cutting force (7.60 N, 7.65 N, 7.67 N) was observed in BR1 (50% sorghum, 27% rice, 23% teff), while the lowest (6.20 N, 6.25 N, 6.46 N) was seen in BR5 (43% sorghum, 27% rice, 30% teff) on days 1, 2, and 4 of storage, respectively (Table 6).

**FIGURE 5:**
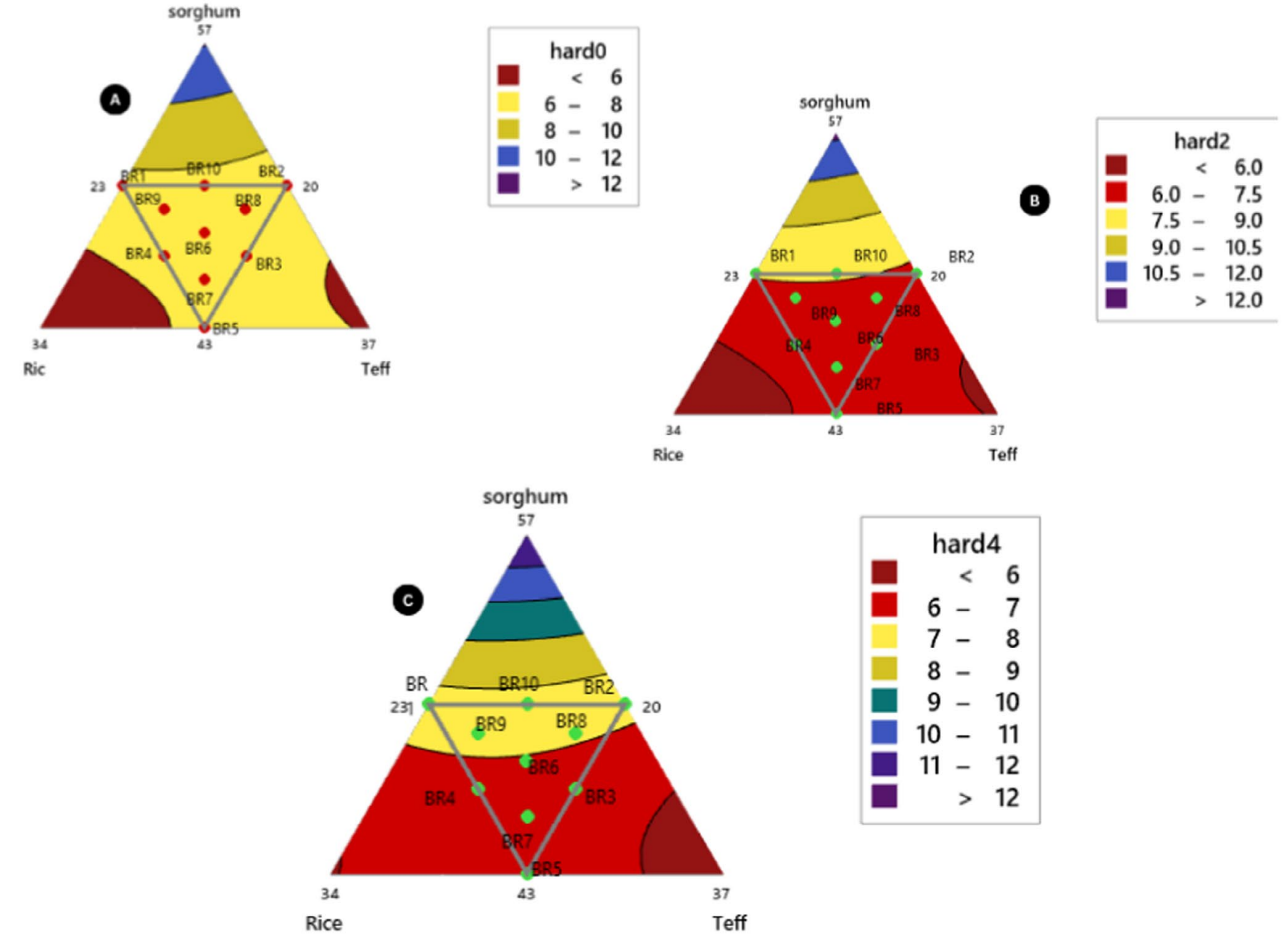
Hardness cutting force at different storage duration periods (A) day‐1 hardness cutting force response, (B) day‐2 hardness cutting force response, (C) day‐4 storage hardness cutting force response.

Analyzing the compressed cutting texture force of stored *injera*, it was found that the texture response varied across the 10 experimental runs. The linear and quadratic interaction between sorghum and teff showed a significant difference (*p* < 0.05), as indicated in Figure 5. Increasing the proportion of sorghum flour in the blend increased the compressed cutting force. However, the quadratic interactions between sorghum and rice, and rice and teff showed no significant difference (*p* < 0.05), as teff and rice flour separately softened the *injera*'s texture during storage. On the second day of storage, the linear and quadratic interactions between sorghum and teff remained significant (*p* < 0.05), while interactions between sorghum and rice, and rice and teff, remained non‐significant (*p* < 0.05).

On the fourth day of storage the texture hardness or compressed cutting forces the linear and quadratic interaction of components sorghum*rice and sorghum*teff significance difference (*p* < 0.05) and non‐significance interaction difference (*p* < 0.05) with rice* teff flour compositions. This means that the compressed cutting force of rice and teff flour *injera* and its constitute affects the softness or rollability of the *injera* product. The result has shown that 100% sorghum *injera* (control 1) compressed cutting force in all the storage periods is higher than all blending ratios and teff flour control *injera*. As compared to sorghum flour *injera* composite flour influences the staling initiation during storage and supplementation of flaxseed also affects its texture hardness and starch reassociation of baked *injera* starting from cooling. Because flaxseed flour limits moisture loss from the crust and provides good water‐binding capacity for the composite flour *injera* product (Pohjanheimo et al. 2006; Inglett, Chen, and Lee 2013).

(14) $$\begin{array}{c} \text{Texture Hardness Model}=1.08013*\text{sorghum}+0.26232*\text{rice}\\ +0.27470*\text{teff}–0.02793*\text{sorghum}*\text{rice}–0.02804*\text{sorghum}\\ {}*\text{teff}+0.01640*\text{rice}*\text{teff} \end{array}$$

#### 3.5.4 Instrumental Color (L* Values) Characteristics of Formulated *Injera*

The color of the *injera* product was evaluated based on the commission international Eclairage (CIE) L*a*b* lab color system, where L*, a*, and b* are the lightness/ darkness, redness/ greenness and blueness/yellowness of products, respectively. Table 7 shows the value of 100% sorghum and teff flour *injera* as control and formulated *injera* instrumental color characteristics.

**TABLE 7:** Colorimetry color properties of baked injera products (CON1%–100% sorghum flour injera, CON2%–100% teff flour injera, BR – blending ratio from BR1 up to BR10).

Compositions	L* value	a* value	b* value	Color difference (∆E)	Whiteness index (WI)
CON1	77.89 ± 0.88^a^	2.17 ± 0.14^b^	13.72 ± 0.51^a^	23.39 ± 0.73^a^	73.89 ± 1.02^a^
CON2	71.14 ± 0.52^d^	1.28 ± 0.15^c^	8.78 ± 0.09^cd^	16.08 ± 0.51^d^	69.80 ± 0.51^c^
BR1	72.22 ± 1.13^bcd^	3.39 ± 0.06^a^	9.97 ± 0.10^b^	17.02 ± 1.11^bcd^	70.29 ± 1.09^bc^
BR2	73.89 ± 0.59^b^	3.15 ± 0.09^a^	9.77 ± 0.20^bc^	17.06 ± 0.69^bcd^	69.82 ± 0.21^c^
BR3	71.77 ± 0.84^cd^	3.19 ± 0.14^a^	9.58 ± 0.14^bcd^	16.54 ± 0.83^cd^	70.02 ± 0.85^c^
BR4	72.27 ± 0.67b^cd^	3.41 ± 0.05^a^	9.59 ± 0.08^bcd^	16.35 ± 0.21^cd^	70.43 ± 0.58^bc^
BR5	71.59 ± 0.21^cd^	3.10 ± 0.16^a^	8.61 ± 0.57^d^	18.59 ± 0.59^b^	72.33 ± 0.72^ab^
BR6	71.64 ± 0.87^cd^	3.39 ± 0.09^a^	9.35 ± 0.28^bcd^	16.38 ± 0.86^cd^	69.94 ± 0.50^c^
BR7	72.39 ± 0.58^bcd^	3.37 ± 0.06^a^	9.47 ± 0.05^cd^	17.14 ± 0.59^bcd^	70.62 ± 0.32^bc^
BR8	73.02 ± 0.32^bcd^	3.20 ± 0.02^a^	9.70 ± 0.07^bc^	17.79 ± 0.32^bcd^	71.15 ± 0.16^bc^
BR9	72.36 ± 0.51^bcd^	3.25 ± 0.35^a^	9.03 ± 0.91^bcd^	17.09 ± 0.49^bcd^	70.73 ± 0.45^bc^
BR10	73.32 ± 0.37^bc^	3.22 ± 0.05^a^	9.32 ± 0.21^bcd^	18.05 ± 0.36^bc^	71.55 ± 0.22^bc^

*Note:* The alphabetic symbols represent statistical groupings for the colorimetry color properties (L*, a*, b*, ∆E, and Whiteness Index) of the baked injera products. These symbols were derived from statistical analyses (likely ANOVA) that compare the color properties of the different compositions (CON1, CON2, BR1, BR2, etc.). (a) typically represents the highest or most distinct value for a given color property (e.g., L*, a*, or b*). (b), (c), and (d) represent other groups, showing that the formulations within each group have similar values for that particular property but differ from other groups.

**FIGURE 6:**
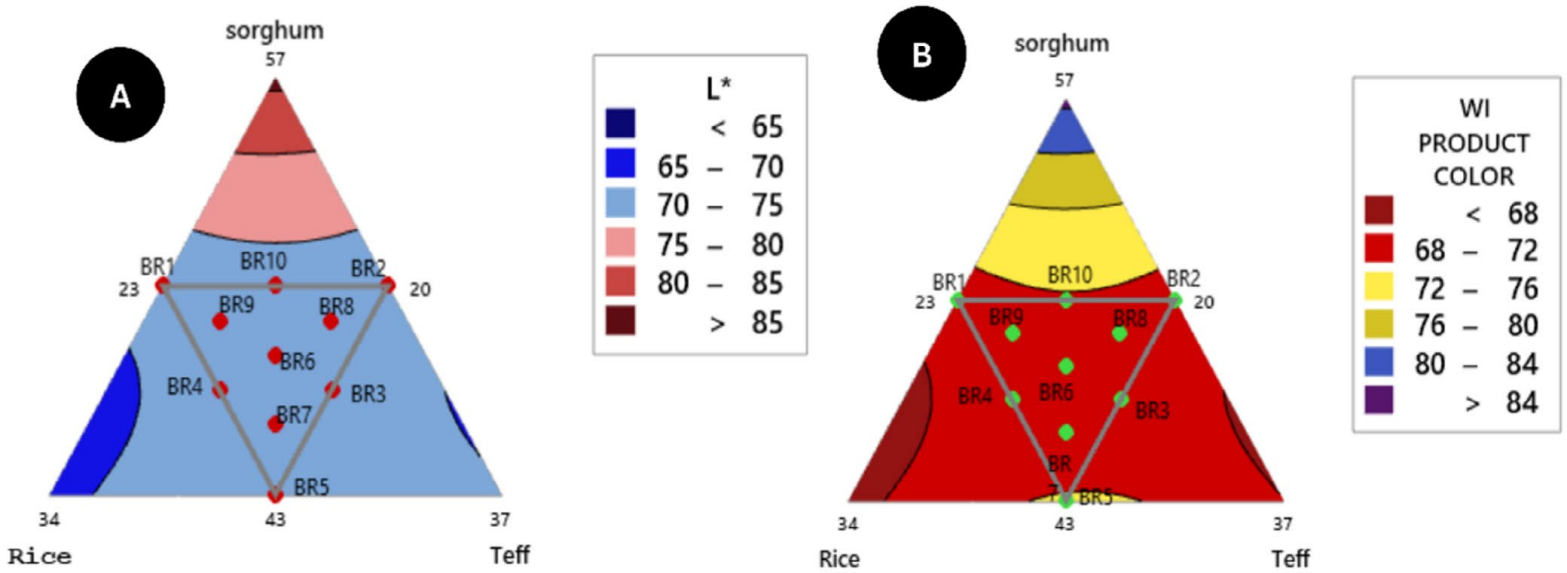
Mixture contour plot of composite flour (A)lightness and (B) whiteness interaction.

Color values of composite flour *injera* products were non‐significant difference (*p* < 0.05) in the linear model but significant difference (*p* < 0.05) sorghum with rice, and rice with teff interaction in L* values (lightness) between the formulated *injera* samples as the blending ratio varies. The mean L* values (lightness) ranged from 71.59 to 73.89. A higher L* value means obtained lighter *injera* whereas, a lower L* value means obtained darker *injera*. The highest L* value (73.89) was found in the blending ratios BR2 with a composition of (50% sorghum, 20% rice, and 30% teff), and the lowest L* value (71.59) was found in BR5 (43% sorghum, 27% rice, 30% teff) blending, but 100% pure sorghum and teff *injera* whiteness L* value 77.89 and 71.14 respectively and (R^2^ = 86.35%). In this study, from the contour plot component interaction observed in Figure 6, the higher the composition of teff flour in the mixture, the higher the L* value observed. Whereas, a higher composition of teff and brown rice flour showed a lower L* value.

(15) $$\begin{array}{c} L*\text{value}=4.57238*\text{sorghum}+0.74273*\text{rice}+2.00206\\ {}*\text{teff}–0.10294*\text{sorghum}*\text{rice}–0.12203*\text{sorghum}*\text{teff}\\ +0.09148*\text{rice}*\text{teff} \end{array}$$

It indicates that the addition of teff and brown rice produced the lowest increase in the lightness (whiteness) of *injera* samples while the addition of sorghum produced the highest increase in the L* values of the samples. The color difference (∆E) value of *injera* products ranged from lowest (BR4) 16.35 to highest (BR5)18.59, the control of sorghum and teff flour *injera* detected 23.39 and 16.08 respectively.

(16) $$\begin{array}{c} \mathrm{LAB}\;\text{Color difference}\;\left(∆E\right)=3.95466*\text{sorghum}+0.22165\\ {}*\text{rice}+1.56779*\text{teff}–0.10081\text{sorghum}*\text{rice}–0.12195\\ {}*\text{sorghum}*\text{teff}+0.08629*\text{rice}*\text{teff} \end{array}$$

(17) $$\begin{array}{c} \text{Whiteness index}\;\left(\mathrm{WI}\right)=4.69458*\text{sorghum}+0.66851\\ {}*\text{rice}+1.68724*\text{teff}-0.10807*\text{sorghum}*\text{rice}\\ –0.12157*\text{sorghum}*\text{teff}+0.10377*\text{rice}*\text{teff} \end{array}$$

The whiteness of sorghum flour is greater than teff flour using lab colorimetry analysis. The whiteness index value also ranged from 69.82–72.33, with the lowest value BR2 and highest value BR5 from the blending ratio variations. The whiteness index values of the two controls (100% sorghum and teff flour *injera*) were 73.89 and 69.80, respectively.

### 3.6 Sensory Evaluation of Baked *Injera*

**FIGURE 7:**
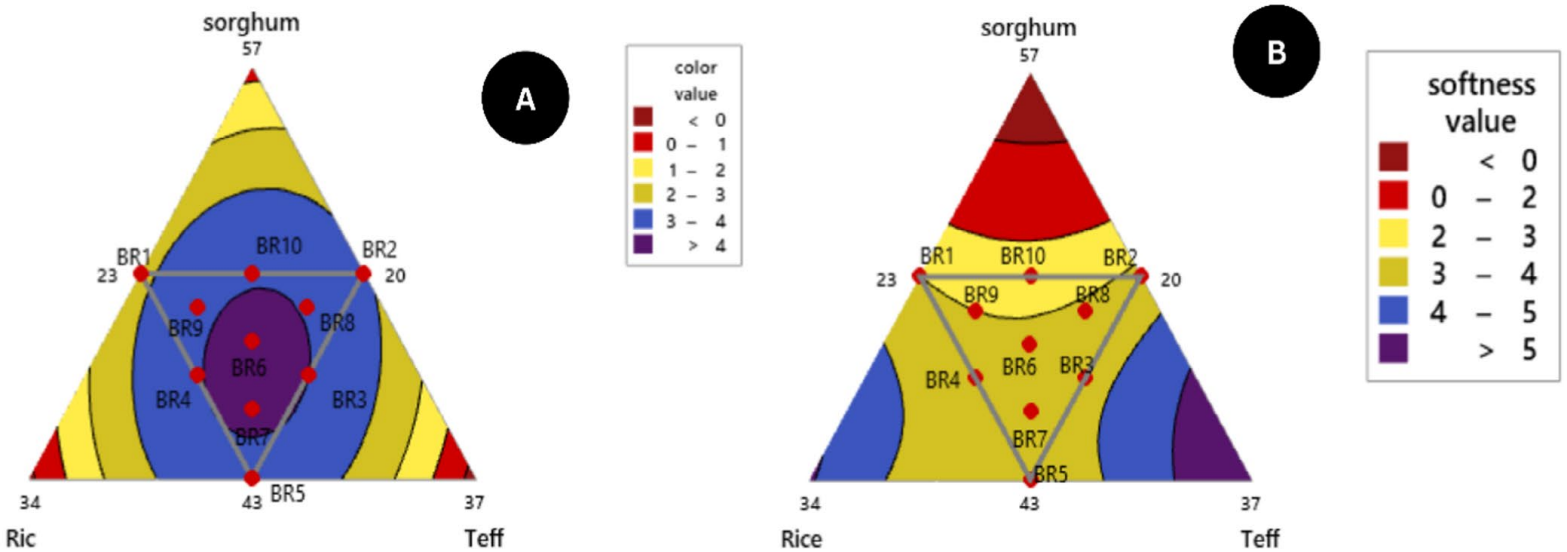
Contour plot of (A) color and (B) softness interaction response.

Sensory evaluation of *injera* produced from sorghum, rice, and teff flour at different blending mixture ratios is presented in Table 8. The color of the baked *injera* ranged from 2.70–4.20 and was obtained using a five‐point hedonic scale. There was a non‐significant difference in color (*p* < 0.05) in the evaluation of the panelists among the 10 experimental runs except *injera* made from BR1 and BR2. All the blending were obtained or accepted greater than 4 (like moderately) by the panelists except from BR1(2.7) and BR2 (3.0). The contour plot (Figure 7) shows that those formulations having low sorghum, rice, and teff flour in the composition had shown a relatively maximum color value. The study shows that white sorghum has a good coloring effect for whiter *injera* baking.

**TABLE 8:** Sensory acceptability of fresh injera using a five‐point hedonic scale (CON1%‐100% sorghum flour injera, CON2%–100% teff flour injera, BR—blending ratio from BR1 up to BR10).

Composition	Color	Softness	Rollability	Taste	Eye distribution	Mouthfeel	OAA
CON1	3.45 ± 0.06^abcd^	2.40 ± 0.09^e^	2.32 ± 0.16^e^	2.45 ± 0.18^g^	2.11 ± 0.02^h^	2.13 ± 0.03^g^	2.20 ± 0.05^g^
CON2	3.21 ± 0.09^bcd^	3.83 ± 0.07^a^	3.83 ± 0.17^ab^	3.74 ± 0.05^b^	3.47 ± 0.04^c^	2.13 ± 0.03^bc^	3.66 ± 0.02^cd^
BR1	2.70 ± 0.08^d^	3.00 ± 0.03^d^	3.00 ± 0.00^d^	2.75 ± 0.05^f^	2.50 ± 0.01^g^	2.50 ± 0.05^f^	2.65 ± 0.05^f^
BR2	3.00 ± 0.03^cd^	3.24 ± 0.21^bc^	3.10 ± 0.21^d^	4.30 ± 0.10^a^	4.11 ± 0.01^a^	4.20 ± 0.10^a^	4.11 ± 0.01^ab^
BR3	4.00 ± 0.15^ab^	3.50 ± 0.05^ab^	3.68 ± 0.25^bc^	3.45 ± 0.06^cd^	2.77 ± 0.03^ef^	3.45 ± 0.18^cd^	3.68 ± 0.05^cd^
BR4	4.00 ± 0.29^ab^	3.47 ± 0.04^bc^	4.20 ± 0.01^a^	3.50 ± 0.05^c^	3.85 ± 0.13^ab^	3.72 ± 0.06^b^	3.98 ± 0.20^b^
BR5	3.75 ± 0.35^abc^	3.72 ± 0.16^cd^	4.21 ± 0.00^a^	4.30 ± 0.03^a^	4.10 ± 0.20^a^	3.87 ± 0.12^e^	4.22 ± 0.04^a^
BR6	4.10 ± 0.62^a^	3.24 ± 0.06^cd^	3.50 ± 0.04^bc^	3.27 ± 0.02^e^	3.12 ± 0.07^d^	3.00 ± 0.01^d^	3.11 ± 0.02^e^
BR7	3.96 ± 0.35^ab^	3.50 ± 0.05^bc^	4.20 ± 0.05^a^	3.75 ± 0.03^b^	3.64 ± 0.07^bc^	3.40 ± 0.10^d^	3.75 ± 0.01^c^
BR8	4.20 ± 0.17^a^	3.21 ± 0.03^cd^	3.38 ± 0.09^cd^	3.51 ± 0.04^c^	3.04 ± 0.03^d^	3.30 ± 0.07^d^	3.50 ± 0.05^d^
BR9	3.85 ± 0.23^abc^	3.03 ± 0.14^d^	3.34 ± 0.11^cd^	3.32 ± 0.04^de^	3.00 ± 0.10^de^	2.50 ± 0.13^f^	2.75 ± 0.06^f^
BR10	3.81 ± 0.39^abc^	2.50 ± 0.03^e^	2.50 ± 0.20^e^	2.60 ± 0.07^fg^	2.50 ± 0.14^fg^	2.33 ± 0.02^fg^	2.33 ± 0.07^g^

*Note:* The alphabetic symbols represent statistical groupings for the sensory acceptability parameters of the fresh injera products (Color, Softness, Rollability, Taste, Eye Distribution, Mouthfeel, and Overall Acceptability) based on the five‐point hedonic scale. These groupings are the result of statistical tests (likely ANOVA) that determine which formulations differ significantly in sensory characteristics. The (a) group typically represents the formulations with the most favorable sensory characteristics for a specific attribute. (b), (c), and so on represent other groups with either similar or significantly lower sensory scores compared to the group marked with (a).

Sorghum and teff flour *injera* as a control scored 3.45 and 3.21, respectively that implies without blending sorghum flour *injera* shows a better color than teff. The color interaction of composite flour *injera* products was significantly different (*p* < 0.05) in the linear model between each component and quadratic model sorghum with rice interaction, and highly significant difference (*p* < 0.05) in a quadratic model of color interaction between sorghum with rice and rice with teff.

The softness value of *injera* reported by the panelists is found to be 2.50–3.72 using a five‐point hedonic scale assessment. Softness response varies among the 10 formulations of *injera* at a significance difference (*p* < 0.05) found the minimum (2.50) and the maximum response (3.72) in BR10 (50% sorghum, 23.5% rice, 26.5% teff) and BR5 (43% sorghum, 27% rice, 30% teff), respectively. Almost all the blended ratios are shown by the panelists neither like nor dislike. There is an increasing trend of liking the softness when the composition of teff and rice is increased (Cherie et al. 2018). This is in agreement with the findings of (Yetneberk, Rooney, and Taylor 2005) who reported that sorghum *injera* is more friable, staling, and softness texture acceptance decreased when the composition of sorghum is increased. Teff and rice flour composition increases as softness increases and inversely sorghum flour composition increases as injera softness decreases (Figure 7). The softness of *injera* is dominant in teff followed by rice flour.

The rollability value of *injera*, as reported by the panelists, ranges from 2.50 to 4.21. The panelists' evaluations significantly varied among the 10 different *injera* formulations (*p* < 0.05). The lowest rollability value (2.50) was recorded for BR10 (50% sorghum, 23.5% rice, 26.5% teff), while the highest value (4.21) was noted for BR5 (43% sorghum, 27% rice, 30% teff). Most of the *injera* blends were rated by the panelists as neither liked nor disliked to moderately liked. *Injera* made from sorghum and teff flour alone scored 2.32 and 3.83, respectively, indicating that *injera* made solely from sorghum flour had poorer rollability compared to composite sorghum‐based *injera* products.

**FIGURE 8:**
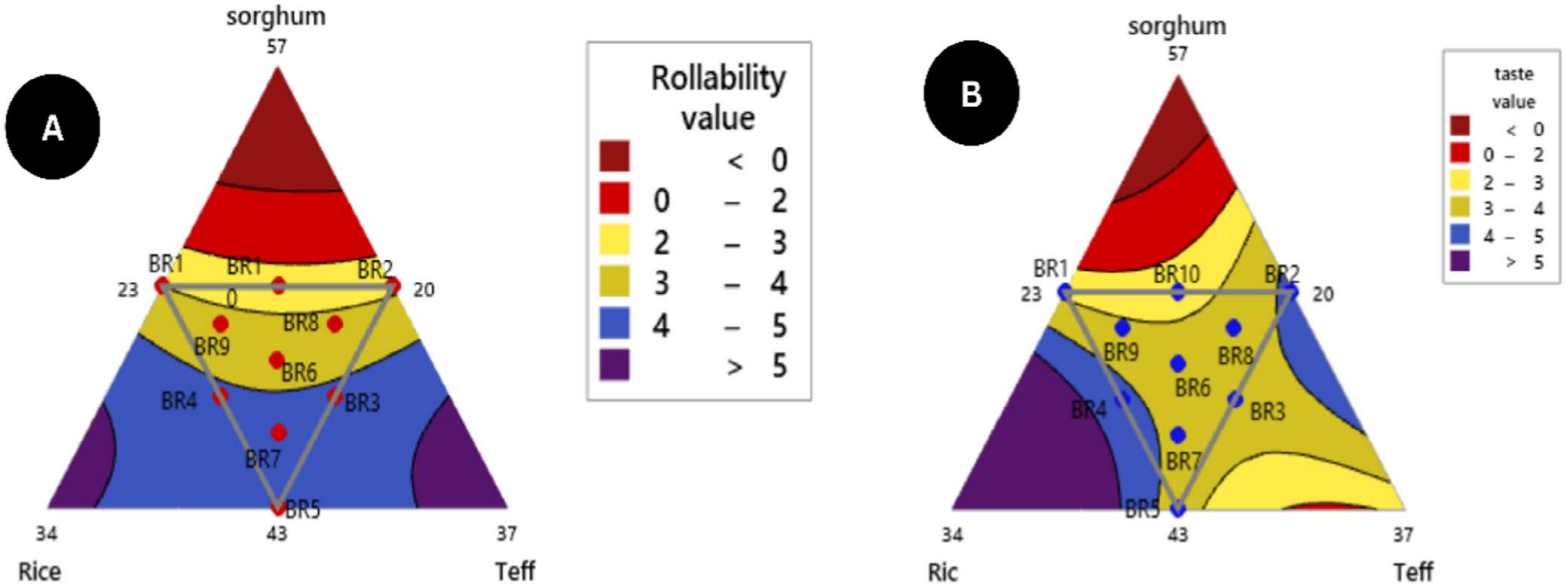
Contour plot of (A) rollability and (B) taste component interaction response.

According to (Yetneberk, Rooney, and Taylor 2005) poor rollability and more dryness were the main drawbacks of sorghum injera. Teff and rice flour composition increases as rollability increases and inversely sorghum flour composition increases as injera rollability decreases (Figure 8).

The taste response of the blending formulated *injera* was determined in a range of 2.60–4.30 using a five‐point hedonic scale. Sorghum and teff flour *injera* as control scored 2.75 and 3.74, respectively. The panelist results showed that except for sorghum flour control, all of the experimental runs *injera* scored above average (2.5) between neither like nor dislike and like moderately. The relative taste acceptability of maximum (4.30) scores was observed in BR2 and BR5 and minimum (2.60) scores were observed in BR10. Mixture contour plot and blending ratios have a significant impact on the taste result of all composite flour *injera* at (*p* < 0.05) (Figure 8).

The mixture contour plot and the regression equation 21 showed that the addition of rice flour resulted in the highest taste and sorghum flour negatively affected product taste. As the percentage of sorghum flour composition reduced whereas, teff and rice flour composition raised the sensory acceptability of taste response was better.

The eye distribution of the formulated *injera* ranged from 2.50–4.11 according to panelists using a five‐point hedonic scale evaluation. The eye distribution of formulated *injera* sensory scales is higher than the average (2.5) and in comparison, relatively composition BR2 and BR5 have scored (above 4) moderately like. The contour plot interaction (Figure 8) shows that the eyes distribution of injera was more evenly distributed. It depends on second fermentation gas bubbles formation for escaping of carbon dioxide (CO_2_) (Bultosa, Hall, and Taylor 2002; Girma, Bultosa, and Bussa 2013). The previous researchers (Abewa and Abay 2020; Kefale 2020) the eye distribution of composite flour *injera* ranged (from 1.75–3.93) using a five‐point hedonic scale and the present result agreed with those findings.

**FIGURE 9:**
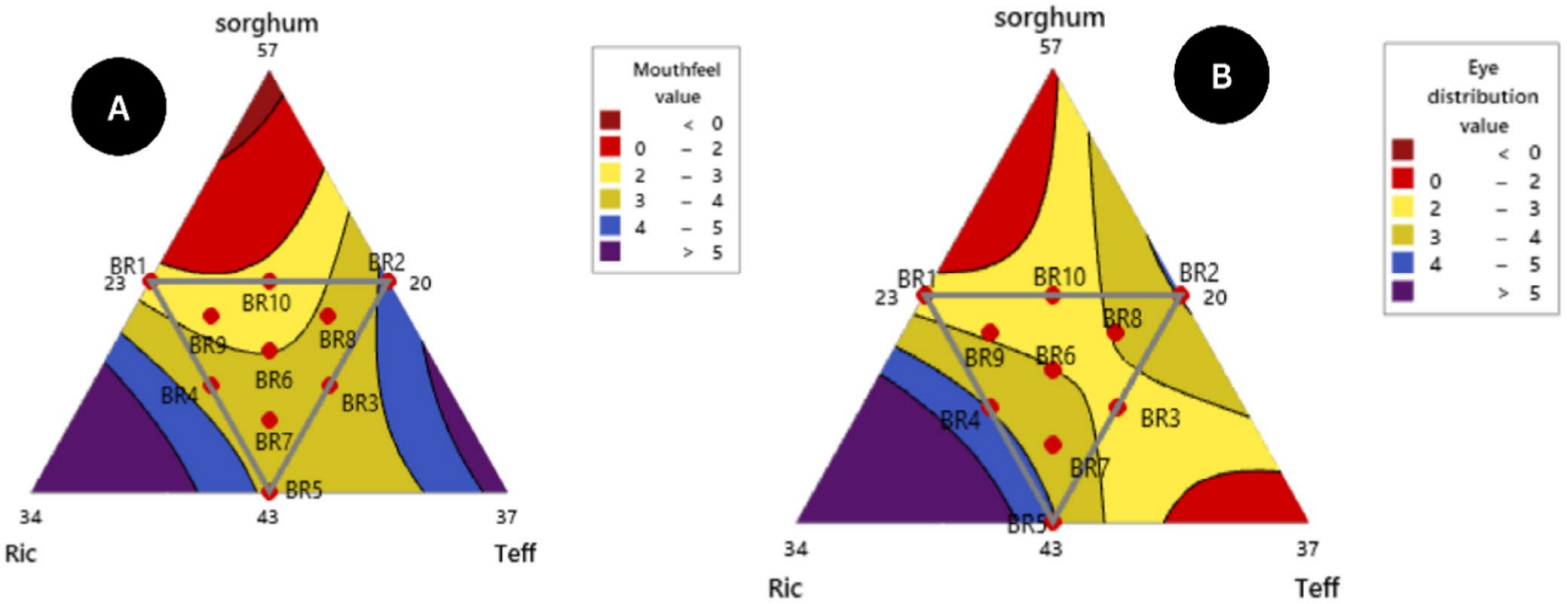
Contour plot of (A) mouthfeel and eye distribution (B) interaction response.

The mouthfeel response of the blending formulated *injera* was scored in a range of 2.33–4.20 from a five‐point hedonic scale untrained panelist assessment. The lowest mouthfeel responses obtained from the ten blending *injera* products were BR10 (50% S, 23.5% R, 26.5% T) and the highest BR2 (50% S, 20% R, 30% T) respectively. Even though, the proportion of sorghum flour was constant in both higher and lower panelist scores, when the teff composition was raised the sensory acceptability of mouthfeel response was better (Girma, Bultosa, and Bussa 2013). The panelist evaluated the *injera* product under a five‐point hedonic scale scoring neither like nor dislike and moderately like except BR1, BR9, and BR10. The highest (4.20) mouthfeel response of blending *injera* products value is highly significant than that of teff flour *injera* as control (2.13) response. It was also studied by (Abewa and Abay 2020) that the mouthfeel sensory attribute of blended flour injera varied from (2.8 to 4.6). As indicated in Figure 9 and the prediction model of mouthfeel equation coefficients described in Equation 23, the rice composition increased product mouthfeel also increased.

The overall acceptability of the ten *injera* formulations ranged from 2.33 to 4.22, as rated by the panelists. These formulated *injera*s were significantly different (*p* < 0.05) from the sorghum control *injera*, which had an acceptability rating of 2.20. Among the formulations, *injera* made from BR5 (43% sorghum, 23% rice, 30% teff) was moderately liked the most (4.22), while *injera* made from BR10 (50% sorghum, 23.5% rice, 26.5% teff) was moderately disliked the most (2.33). Studies by various researchers on *injera* made from 100% teff flour and composite flour showed overall acceptability ranging from 1.72 to 4.53 on a five‐point hedonic scale (Abraha and Abay 2017; Woldemariam et al. 2019b; Abewa and Abay 2020). The results indicate that *injera* with a higher proportion of teff flour is generally more preferred than *injera* made entirely from sorghum flour.

**FIGURE 10:**
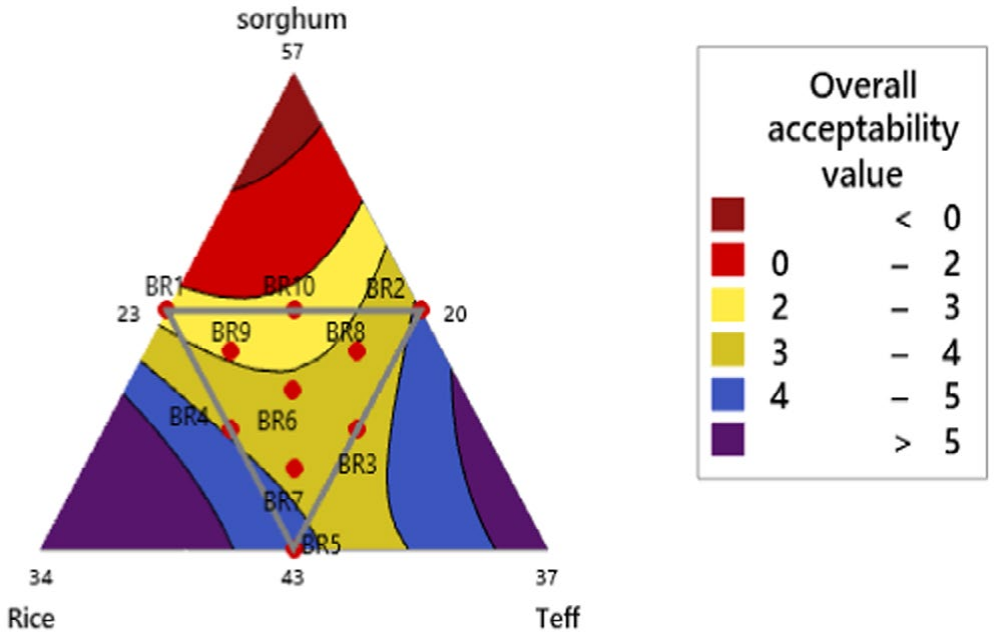
Contour plot of overall acceptability component interaction response.

Figure 10 shows the overall acceptability of composite flour *injera* mixture contour plot interaction response using a five‐point hedonic scale assessment. It also depicts the interaction of rice and teff flour produced an increased responsibility for the overall acceptability of injera samples. There was a significant difference (*p* < 0.05) in the linear and quadratic models of overall acceptability sensory quality response in the interaction of formulated *injera* components sorghum with rice, sorghum with teff, and rice with teff flour (Table 9).

**TABLE 9:** *p* values, equations, and correlation values of sensory attributes.

Parameters	*R* ^2^	Linear *p* value	Quadratic *p* value	Equations	Equation number
Color	93.24	0.050	0.014	Color equation=−0.95293*SF–2.66969*RF–2.84463*TF+0.05061*SF*RF+0.06004*SF*TF+0.08132*RF*T	(18)
Softness	99.32	0.000	0.001	Softness=1.05680*SF+0.32464*RF+0.75272*TF+0.02905*SF*RF+0.01824*SF*RF–0.04899*RF*TF	(19)
Rollability	98.26	0.025	0.013	Rollability=−1.72771*SF−0.44666*RF–0.03892*TF+0.05442*SF*RF+0.04360*SF*TF–0.03422*RF*TF	(20)
Taste	98.36	0.001	0.001	Taste equation=−1.48316*SF+4.40490*RF–2.25797*TF−0.03836*SF*RF+0.09473*SF*TF–0.07602*RF*TF	(21)
Eye distribution	97.78	0.002	0.003	Eyedistribution=0.00814*SF+5.63982*RF–2.05091*TF−0.09867*SF*RF+0.05628*SF*TF–0.05560*RF*TF	(22)
mouthfeel	98.25	0.002	0.004	Mouthfeel=−0.59787*SF+4.21664*RF–0.17590*TF−0.04994*SF*RF–0.03785*SF*TF–0.08625*RF*TF	(23)
OAA	98.05	0.003	0.006	Overall acceptability=−0.78186SF+3.93905RF–0.09736TF−0.04022SF*RF–0.03976SF*TF–0.08668RF*TF	(24)

Abbreviation: OAA = overall acceptability.

## 4 Conclusion

This study has successfully examined the physicochemical and sensory qualities of injera produced from blends of sorghum, rice, teff, and a flaxseed flour supplement. The research confirmed the initial hypothesis that incorporating flaxseed flour other grain flours which can improve the overall quality and acceptability of injera by enhancing its sensory attributes and overall acceptability profile. The optimal combination of grain ratios, along with flaxseed, positively influenced key characteristics such as texture, taste, softness, and mouthfeel. Moreover, flaxseed flour contributed to favorable physicochemical properties, enhancing the structural integrity and storage stability of the injera. These findings highlight the potential of flaxseed, rice, and sorghum as a functional ingredient and can be effectively integrated into traditional teff grain‐based injera to without compromising its sensory appeal. As a result, flaxseed flour can be recommended for use in both household and industrial production to improve the health benefits and marketability of injera.

## Author Contributions

**Moges Amtataw:** conceptualization, methodology, investigation, software. **Estifanos Kassahun:** conceptualization, methodology, investigation, software. **Solomon Tibebu:** validation, formal analysis, supervision. **Tadele Andargie:** writing – review and editing, writing – original draft, resources. **Takele Ayanaw:** visualization, data curation. **Agimassie Agazie:** data curation. **Mesfin Wogayehu:** conceptualization, methodology, investigation, software. **Abebaw Teshome:** validation, formal analysis, supervision. **Sadik Jemal:** conceptualization, methodology, investigation, software. **Deginet Teferi:** validation, formal analysis, supervision.

## Conflicts of Interest

The authors declare no conflicts of interest.

## Data Availability

The data that support the findings of this study are available on request from the corresponding author.
